# Activation of epidermal growth factor receptor signaling mediates cellular senescence induced by certain pro‐inflammatory cytokines

**DOI:** 10.1111/acel.13145

**Published:** 2020-04-22

**Authors:** Dongsheng Shang, Danlin Sun, Chunyan Shi, Jun Xu, Mingxiang Shen, Xing Hu, Hanqing Liu, Zhigang Tu

**Affiliations:** ^1^ School of Pharmacy Jiangsu University Zhenjiang China; ^2^ Institute of Life Sciences Jiangsu University Zhenjiang China; ^3^ Obstetrics and Gynecology Department Affiliated Hospital of Jiangsu University Zhenjiang China; ^4^ Department of Cognitive Neurology China National Clinical Research Center for Neurological Diseases (NCRC‐ND) Beijing Tiantan Hospital Capital Medical University Beijing China

**Keywords:** EGFR, HUVEC, IMR90, pro‐inflammatory cytokine, Ras signaling, senescence

## Abstract

It is well established that inflammation in the body promotes organism aging, and recent studies have attributed a similar effect to senescent cells. Considering that certain pro‐inflammatory cytokines can induce cellular senescence, systematically evaluating the effects of pro‐inflammatory cytokines in cellular senescence is an important and urgent scientific problem, especially given the ongoing surge in aging human populations. Treating IMR90 cells and HUVECs with pro‐inflammatory cytokines identified six factors able to efficiently induce cellular senescence. Of these senescence‐inducing cytokines, the activity of five (namely IL‐1β, IL‐13, MCP‐2, MIP‐3α, and SDF‐1α) was significantly inhibited by treatment with cetuximab (an antibody targeting epidermal growth factor receptor [EGFR]), gefitinib (a small molecule inhibitor of EGFR), and EGFR knockdown. In addition, treatment with one of the senescence‐inducing cytokines, SDF‐1α, significantly increased the phosphorylation levels of EGFR, as well as Erk1/2. These results suggested that pro‐inflammatory cytokines induce cellular senescence by activating EGFR signaling. Next, we found that EGF treatment could also induce cellular senescence of IMR90 cells and HUVECs. Mechanically, EGF induced cellular senescence via excessive activation of Ras and the Ras‐BRaf‐Erk1/2 signaling axis. Moreover, EGFR activation induced IMR90 cells to secrete certain senescence‐associated secretory phenotype factors (IL‐8 and MMP‐3). In summary, we report that certain pro‐inflammatory cytokines induce cellular senescence through activation of the EGFR‐Ras signaling pathway. Our study thus offers new insight into a long‐ignored mechanism by which EGFR could regulate cellular senescence and suggests that growth signals themselves may catalyze aging under certain conditions.

## Graphical abstract

Pro‐inflammatory cytokines were screened, and five of these cytokines were identified to induce cellular senescence by activating EGFR‐Ras signaling pathway, a long‐ignored mechanism by which EGFR could regulate cellular senescence. These results suggest that growth signals themselves may catalyze aging under certain conditions.

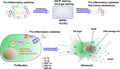

**Abbreviations**
53BP1: p53‐binding protein 1
bFGF: Basic fibroblast growth factor
CXCL12: C‐X‐C motif chemokine ligand 12
CXCR4: C‐X‐C chemokine receptor type 4
CXCR7: C‐X‐C chemokine receptor type 7
DDR: DNA damage response
EGF: epidermal growth factor
EGFR: epidermal growth factor receptor
ELISA: enzyme‐linked immunosorbent Assay
ERK: extracellular signal‐regulated kinase
GM‐CSF: granulocyte‐macrophage colony‐stimulating factor
GRO‐α: growth‐regulated oncogene‐alpha
HUVECs: human umbilical vein endothelial cells
IF: immunofluorescence
IGF‐BP7: insulin‐like growth factor binding protein 7
IL‐13: interleukin‐13
IL‐15: interleukin‐15
IL‐1β: interleukin‐1 beta
IL‐6: interleukin‐6
IL‐7: interleukin‐7
KGF: keratinocyte growth factor
MCP‐2: monocyte chemoattractant protein‐2
MCP‐3: monocyte chemoattractant protein‐3
MEK: mitogen‐activated protein kinase kinase
MIP‐1α: macrophage inflammatory protein‐1 alpha
MIP‐3α: macrophage inflammatory protein‐3 alpha
MMP‐3: matrix metalloproteinase‐3
NSCLC: non‐small‐cell lung cancer
OBIS: oncogene‐induced senescence
OPG: osteoprotegerin
RIS: Ras‐induced senescence
SAHF: senescence‐associated heterochromatic foci
SASP: senescence‐associated secretory phenotype
SA‐β‐gal: senescence‐associated β‐galactosidase
SDF‐1α: stromal cell‐derived factor‐1 alpha
TGFβ1: transforming growth factor beta 1
VEGF: vascular endothelial growth factor
WB: Western blotting
γH2A.X: Phospho‐H2A.X(Ser139)

## 1 INTRODUCTION

Improvements in living and medical conditions over the past several decades have led to increasing numbers of elderly populations worldwide, and a growing demographic problem in many countries that makes delaying aging and improving health in the elderly an important scientific issue. According to geroscience, aging and age‐related chronic diseases share seven evolutionarily conserved mechanistic pillars: inflammation, stem cell regeneration, macromolecular damage, stress, proteostasis, metabolism, and epigenetics (Kennedy et al., 2014). Among these, inflammation plays a central role and also closely interacts with the other six mechanisms (Franceschi, Garagnani, Parini, Giuliani, & Santoro, 2018). Indeed, levels of pro‐inflammatory cytokines tend to increase with age (Franceschi et al., 2000; Shaw, Goldstein, & Montgomery, 2013), and many have been strongly implicated in geriatric diseases, such as skeletal aging (Yu et al., 2014), diabetes (Mauer et al., 2014; Yan et al., 2014), and Alzheimer's disease (Perry, Cunningham, & Holmes, 2007). More importantly, Furman et al. (2017) found that expressions of inflammasome gene modules are higher in older adults compared with young controls and are associated with shorter longevity.

Cellular senescence is a particular type of cell cycle arrest involved in multiple physiological and pathological processes, including embryonic development, wound healing, tumor suppression, and organism aging (He & Sharpless, 2017). More importantly, recent studies have indicated that cellular senescence plays a causal role in aging‐related diseases, including atherosclerosis, cardiovascular dysfunction, diabetes mellitus type 2, and Alzheimer's disease (Tchkonia et al., 2010), while clearing senescent cells can delay or even reverse the aging processes in vitro and in vivo (Childs et al., 2017). For example, Bussian et al. (2018) showed that clearance of senescent glial cells prevents tau‐dependent neurodegenerative processes and cognitive decline in mice, while Baker's studies (Baker et al., 2011, 2016) showed that removing p16‐positive senescent cells extends healthy lifespan and prevents aging‐associated disorders, including sarcopenia, cataracts, and loss of adipose tissue in mice. Recently, Baar et al. (2017) revealed that targeted apoptosis of senescent cells restores tissue homeostasis and counteracts chemotherapy‐induced aging, while Xu et al. (2018) reported that transplanting senescent cells into young mice is sufficient to cause persistent physical dysfunction and accelerate aging of the hosts.

The senescence‐associated secretory phenotype (SASP) is one of the most important features of senescent cells (Coppe et al., 2008; Kuilman et al., 2008), and several groups have intensively studied the cell‐autonomous mechanisms for SASP regulation using different cell models and stimuli (Freund, Patil, & Campisi, 2011; Rodier et al., 2009). Importantly, senescent cells secrete various pro‐inflammatory cytokines, including most of those associated with organism aging (Childs et al., 2017), and recent studies showed that pro‐inflammatory cytokines can induce cellular senescence in vitro and even in vivo (Acosta et al., 2013; Hoare & Narita, 2013).

In summary, the accumulated evidence suggests that: 1, the levels of certain inflammatory factors increase with age; 2, certain inflammatory factors can induce cellular senescence in vitro; and 3, cellular senescence may promote organism aging. Based on these accumulated data, there are two questions that the relevant scientific fields need to address with some urgency: 1, to determine senescence‐inducing inflammatory factors through systematic analysis; and 2, to explore the underlying mechanisms by which inflammatory factors induce cellular senescence.

## 2 RESULTS

### 2.1 Six pro‐inflammatory cytokines induce cellular senescence

According to the published literature, we selected a group of pro‐inflammatory cytokines most closely associated with organism aging, aging‐related diseases, and cellular senescence (Freund, Orjalo, Desprez, & Campisi, 2010; Furman et al., 2017; Hoare & Narita, 2013; Mauer et al., 2014; Perry et al., 2007; Yan et al., 2014). For convenience, we chose 21 commercially available cytokines (Table S4) to screen for their abilities to induce cellular senescence.

The human diploid fibroblast strain IMR‐90 (normal human lung fibroblast) is widely used in aging studies as a normal fibroblast strain. Recent studies have demonstrated that senescence of fibroblasts is important in the process of wound healing (Baker et al., 2016). In addition, senescence of vascular endothelial cells may contribute to cardiovascular diseases, such as atherogenesis (Childs et al., 2016). Thus, IMR90 cells and human umbilical vein endothelial cells (HUVECs) were used in the current study, and we hope our findings will not only advance our understanding of the mechanisms of cellular senescence induced by pro‐inflammatory cytokines, but also improve the understanding of the relevant physiological and pathological processes.

**FIGURE 1:**
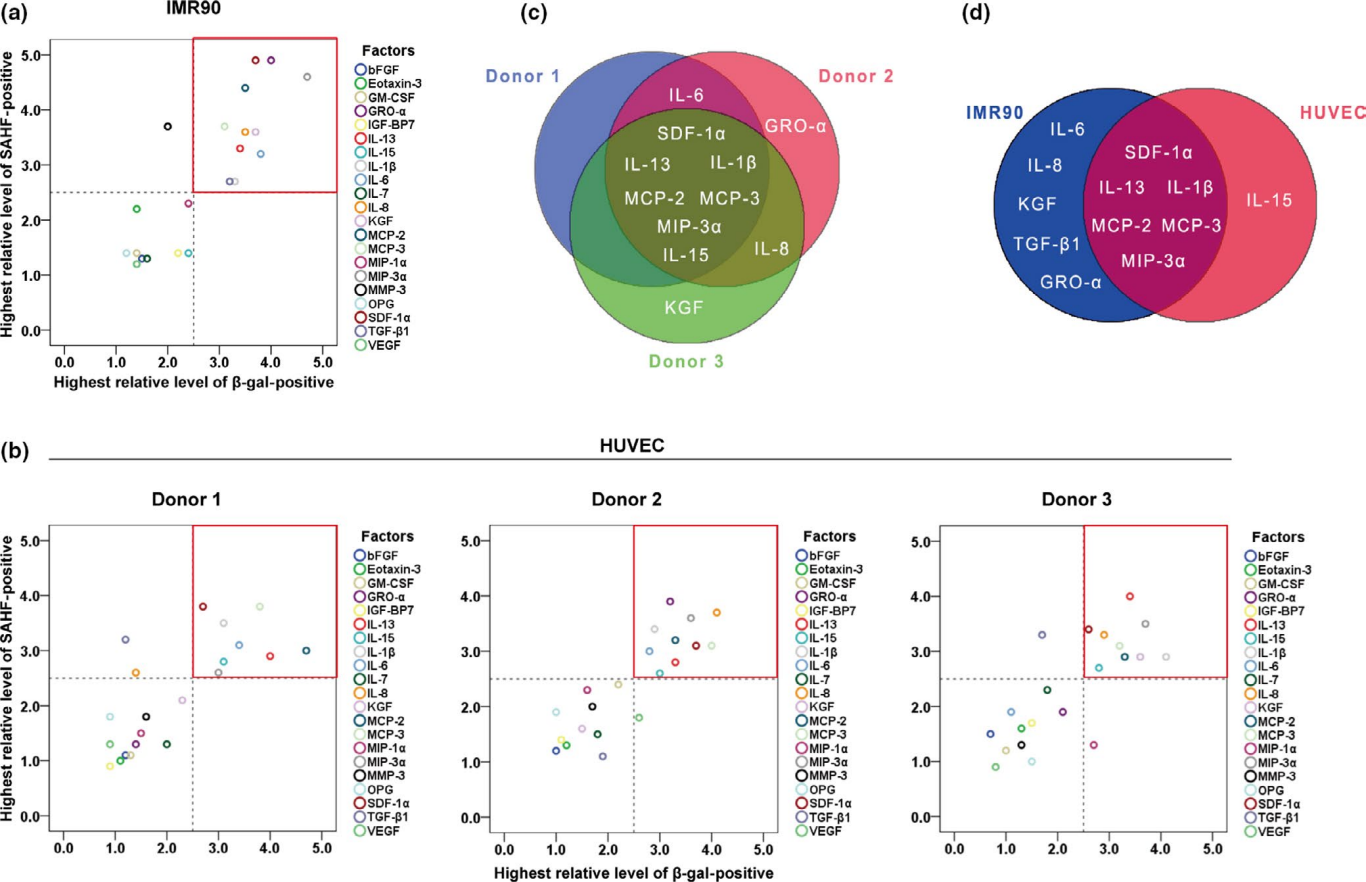
Six pro‐inflammatory cytokines showed activities to induce cellular senescence. (a) Screening of cytokines on IMR90 cells, the values of “Highest relative level of SAHF‐ or β‐gal‐positive” were extracted from Table S5. The area circled by the red box in the upper right corner indicates the senescence inducers. (b) Screening of cytokines on primary cultured HUVECs from three donors, the values of “Highest relative level of SAHF‐ or β‐gal‐positive” were extracted from Table [Link], [Link], [Link]. (c) Venn diagram of three HUVEC strains. (d) Venn diagram of IMR90 cells and HUVECs

The cytokines were individually applied at different concentrations (1, 3, 10, 30, and 100 ng/ml) on IMR90 cells. Formation of senescence‐associated heterochromatic foci (SAHF) and elevated SA‐β‐gal activity are two characteristic biomarkers for senescent cells. In this current study, a cytokine was defined as “positive” in an assay (SAHF or SA‐β‐gal staining) if it dramatically increased the numbers of positive cells (up to 2.5‐fold compared with the basal level) in a dose‐dependent manner (in a certain range). Thus, we defined a cytokine as a “senescence‐inducer” if the cells were positive for both SAHF and SA‐β‐gal staining. As shown in Figure S1, 12 cytokines were defined as positive to induce SAHF formation. Similarly, 11 cytokines were found to dramatically increase SA‐β‐gal activity in cells (Figure S2). We summarized the results from Figures S1 and S2 (see Table S5) and found that 11 cytokines (namely growth‐related oncogene‐α [GRO‐α], interleukin [IL]‐1β, IL‐6, IL‐8, IL‐13, keratinocyte growth factor [KGF], monocyte chemoattractant protein [MCP]‐2, MCP‐3, macrophage inflammatory protein [MIP]‐3α, stromal cell‐derived factor [SDF]‐1α, and transforming growth factor [TGF]‐β1) were positive in both assays (Figure 1a) in IMR90 cells. To further confirm our findings, we screened the original 21 cytokines on three primary HUVEC lines derived from different donors. Similar to IMR90 cells, several cytokines were double positive in both SAHF and SA‐β‐gal assays in different HUVEC strains (Tables [Link], [Link], [Link], Figure 1b). In addition, seven cytokines (namely IL‐1β, IL‐13, IL‐15, MCP‐2, MCP‐3, MIP‐3α, and SDF‐1α) consistently induced senescence in HUVECs (Figure 1c). Based on the results from both cell lines, we identified six cytokines (namely IL‐1β, IL‐13, MCP‐2, MCP‐3, MIP‐3α, and SDF‐1α) that consistently induced cellular senescence (Figure 1d). In addition, we determined the optimal concentrations for each cytokine (10 ng/ml for IL‐13, MCP‐2, MCP‐3, MIP‐3α, and SDF‐1α; 30 ng/ml for IL‐1β) and used the optimal concentrations in the following studies.

### 2.2 Five pro‐inflammatory cytokines induce cellular senescence through activating EGFR signaling

Next, we sought to investigate the mechanisms by which these cytokines induced cellular senescence. Previous studies revealed that certain cytokines, for example IL‐1β and SDF‐1α, can activate EGFR signaling (Porcile et al., 2005). Importantly, Ras is an important downstream effector of EGFR signaling pathways and over‐activated Ras signaling can induce cellular senescence. We therefore hypothesized that certain cytokines might induce cellular senescence through the EGFR‐Ras signaling pathway.

**FIGURE 2:**
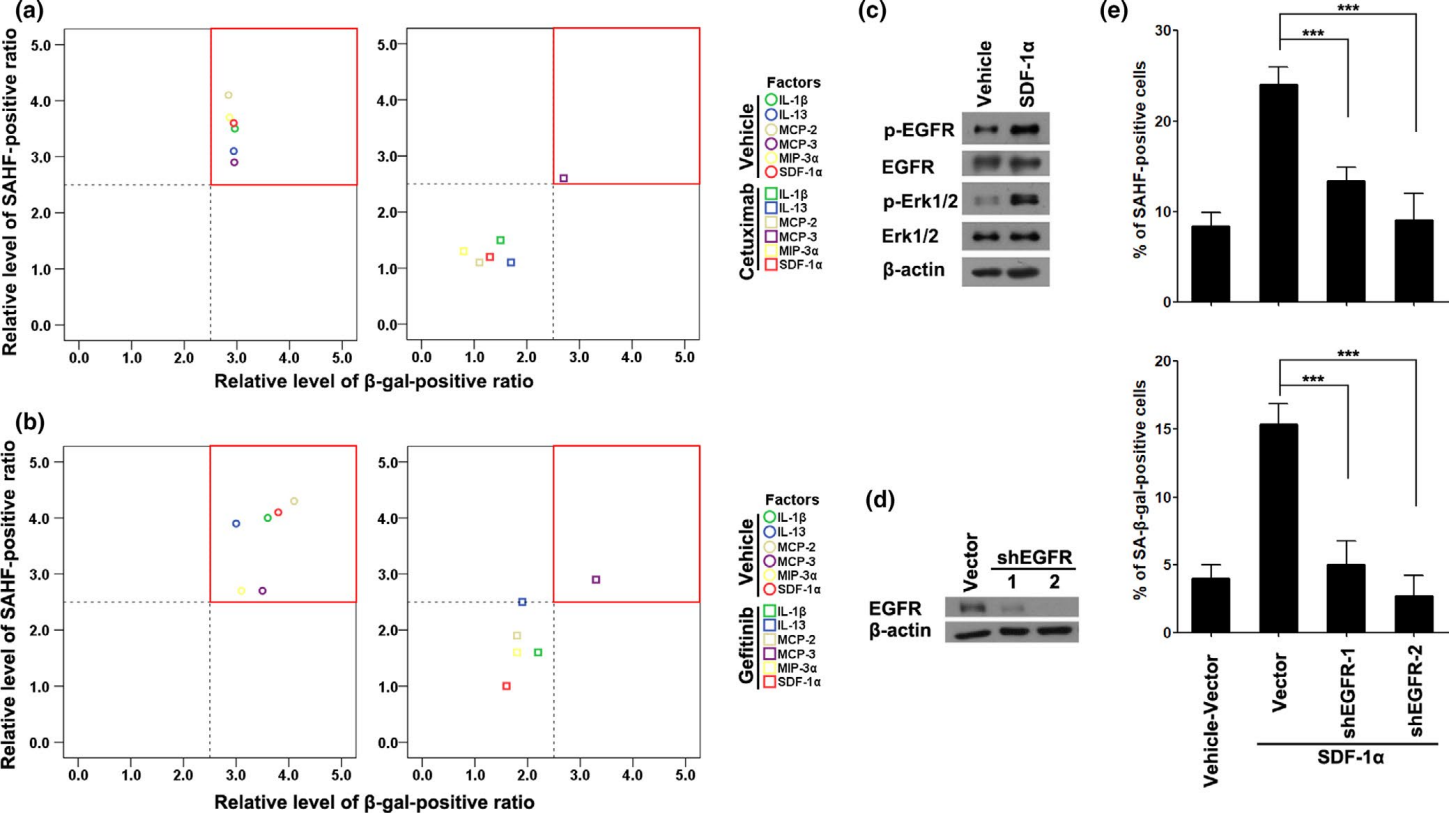
Five pro‐inflammatory cytokines induce cellular senescence through EGFR activation. (a) The effect of cetuximab on senescence‐inducing pro‐inflammatory cytokines; the values of “Relative levels of SAHF‐ or β‐gal‐positive” were extracted from Table S9. The red box in the upper right corner indicates the cytokines inducing senescence under the indicated conditions. (b) The effect of gefitinib on senescence‐inducing pro‐inflammatory cytokines; the values of “Relative levels of SAHF‐ or β‐gal‐positive” were extracted from Table S10. (c) Protein levels of p‐EGFR (Tyr1068), EGFR, p‐Erk1/2 (Thr202/Tyr204), and Erk1/2 in cells with or without SDF‐1α treatment. (d) EGFR protein levels of IMR90 cells with or without EGFR knockdown. (e) Percentages of SAHF‐ or SA‐β‐gal‐positive cells in groups treated as indicated in the relevant figure. Data indicate the mean values calculated from three independent experiments (±*SD*). The Western blots were performed at least three times and the representative pictures are presented

Cetuximab, an antibody targeting EGFR, was first used to test this hypothesis. As shown in Figure S3, cetuximab treatment inhibited the senescence‐inducing activities of five cytokines (IL‐1β, IL‐13, MCP‐2, MIP‐3α, and SDF‐1α), but had no such effect on MCP‐3. The results in Figure S3 are summarized in Table S9 and Figure 2a (for better clarity, we used the same color but different shapes to indicate the same cytokine with and without cetuximab treatment). Consistently, gefitinib, a small molecule inhibitor of EGFR, also dramatically inhibited these five cytokines to induce senescence, but had no effect on MCP‐3 (Figure S4, Table S10, and Figure 2b).

Next, we used SDF‐1α as an example to further examine our hypothesis. As expected, results showed that SDF‐1α treatment significantly increased the phosphorylation levels of EGFR as well as Erk1/2, one of the most important downstream effectors of EGFR activation (Figure 2c). EGFR knockdown dramatically inhibited SDF‐1α‐induced cellular senescence manifested by a decreased percentage of SAHF‐ and SA‐β‐gal‐positive cells (Figure 2d,e). Similarly, EGFR knockdown dramatically inhibited senescence induced by IL‐1β, IL‐13, MCP‐2, and MIP‐3α, but left MCP‐3 alone (Figure S5).

Since EGF is one of the most important ligands of EGFR, we then measured the amounts of EGF secreted by the cells stimulated by the cytokines. To our surprise, treatment with cytokines (IL‐1β or SDF‐1α) did not increase EGF secretion (Figure S6). So far, seven different ligands have been reported to bind mammalian EGFR (Clasadonte, Sharif, Baroncini, & Prevot, 2011). The previous studies have shown that both IL‐1β and SDF‐1α can activate EGFR through c‐Src‐mediated EGFR transactivation (Cheng, Kuo, Lin, Hsieh, & Yang, 2010; Porcile et al., 2005). Therefore, we speculated that EGFR activation by cytokines could occur through an EGF‐independent pathway using other ligands or through a ligand‐independent pathway. Nevertheless, the above results demonstrate that five cytokines (IL‐1β, IL‐13, MCP‐2, MIP‐3α, and SDF‐1α) induce cellular senescence through EGFR activation, and we can use EGF as a tool to activate EGFR in in vitro experiments.

### 2.3 EGF treatment induces cellular senescence in IMR90 cells and HUVECs

**FIGURE 3:**
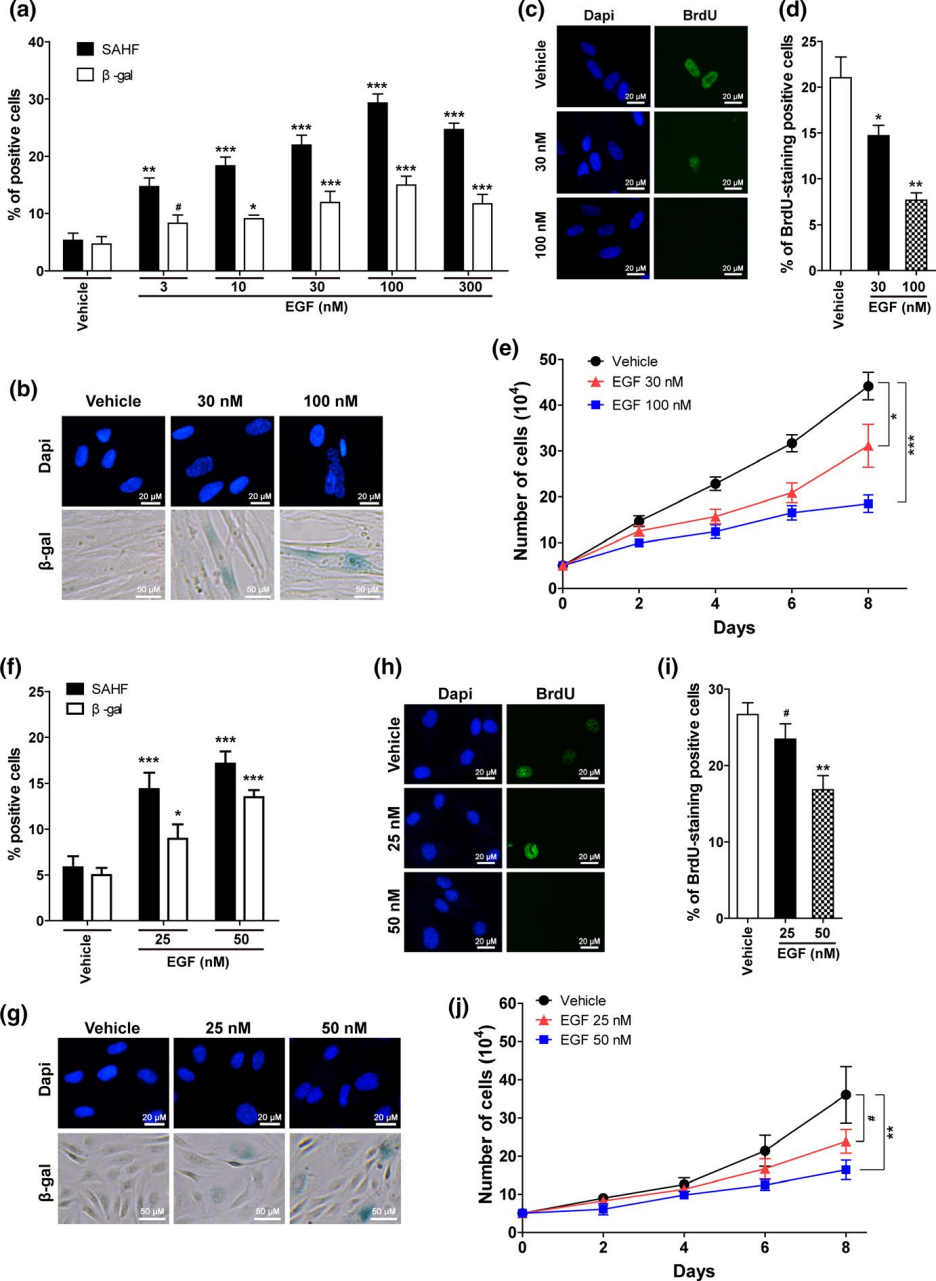
EGF treatment induced senescent phenotypes in IMR90 cells and HUVECs. (a) IMR90 cells were treated with different concentrations of EGF for 3 days, and the percentages of SAHF‐ and β‐gal‐positive cells were analyzed. (b) Representative pictures of DAPI and SA‐β‐gal staining on IMR90 cells with or without EGF treatment at the indicated concentrations for 3 days. (c) Representative pictures of BrdU staining on IMR90 cells treated with or without EGF treatment at the indicated concentrations for 3 days. (d) Statistical analysis of c. (e) The growth curves of IMR90 cells treated with or without EGF treatment at the indicated concentrations. (f) HUVECs were treated with different concentrations of EGF for 3 days, and the percentages of SAHF‐ and β‐gal‐positive cells were analyzed. (g) Representative pictures of DAPI and SA‐β‐gal staining on HUVECs with or without EGF treatment at the indicated concentrations for 3 days. (h) Representative pictures of BrdU staining on HUVECs with or without EGF treatment at the indicated concentrations for 3 days. (i) Statistical analysis of h. (j) The growth curves of HUVECs treated with or without EGF at the indicated concentrations. Data indicate the mean values calculated from three independent experiments (±*SD*)

IMR90 cells were treated with EGF at different concentrations (0, 3, 10, 30, 100, and 300 nM), and as shown in Figure 3a,b, EGF treatment increased the percentages of SAHF‐ and β‐gal‐positive cells in a dose‐dependent manner at concentrations of 100 nM or less. The effects on cell proliferation were also measured using 5‐bromo‐2‐deoxyuridine (BrdU) incorporation assays. As expected, EGF treatment significantly decreased the percentages of BrdU‐positive cells when used at 30 and 100 nM, compared with vehicle‐treated controls (Figure 3c,d). EGF is generally considered to promote cell proliferation; however, our study showed EGF (30 and 100 nM) inhibited growth of IMR90 cells, possibly because the EGF treatment induced cellular senescence (Figure 3e). We also showed that EGF treatment did not induce a significant increase in apoptosis in the IMR90 cells, suggesting that cell growth was not inhibited through inducing apoptosis (Figure S7). We conducted similar experiments on HUVECs. Although we had three HUVEC lines from three donors, we chose the second one since it had the largest number of cells at that stage. As expected, EGF treatment significantly increased the proportions of SAHF‐ and β‐gal‐positive cells in HUVECs (Figure 3f,g). These effects were dose‐dependent, and more importantly, the percentages of positive cells in the 50 nM group were increased to 2.94‐ and 2.74‐fold for SAHF and β‐gal, respectively, compared with the control group. Therefore, according to our previous definition, 50 nM of EGF can significantly induce cellular senescence in HUVECs. Consistently, 50 nM of EGF significantly inhibited cell growth, as manifested by decreased BrdU incorporation (Figure 3h,I) and lower cell numbers, after being cultured for 8 days (Figure 3j). These data demonstrate that EGF treatment inhibits cell growth by inducing cellular senescence in IMR90 cells and HUVECs.

### 2.4 EGF treatment activates signaling pathways involved in Ras‐induced senescence (RIS)

**FIGURE 4:**
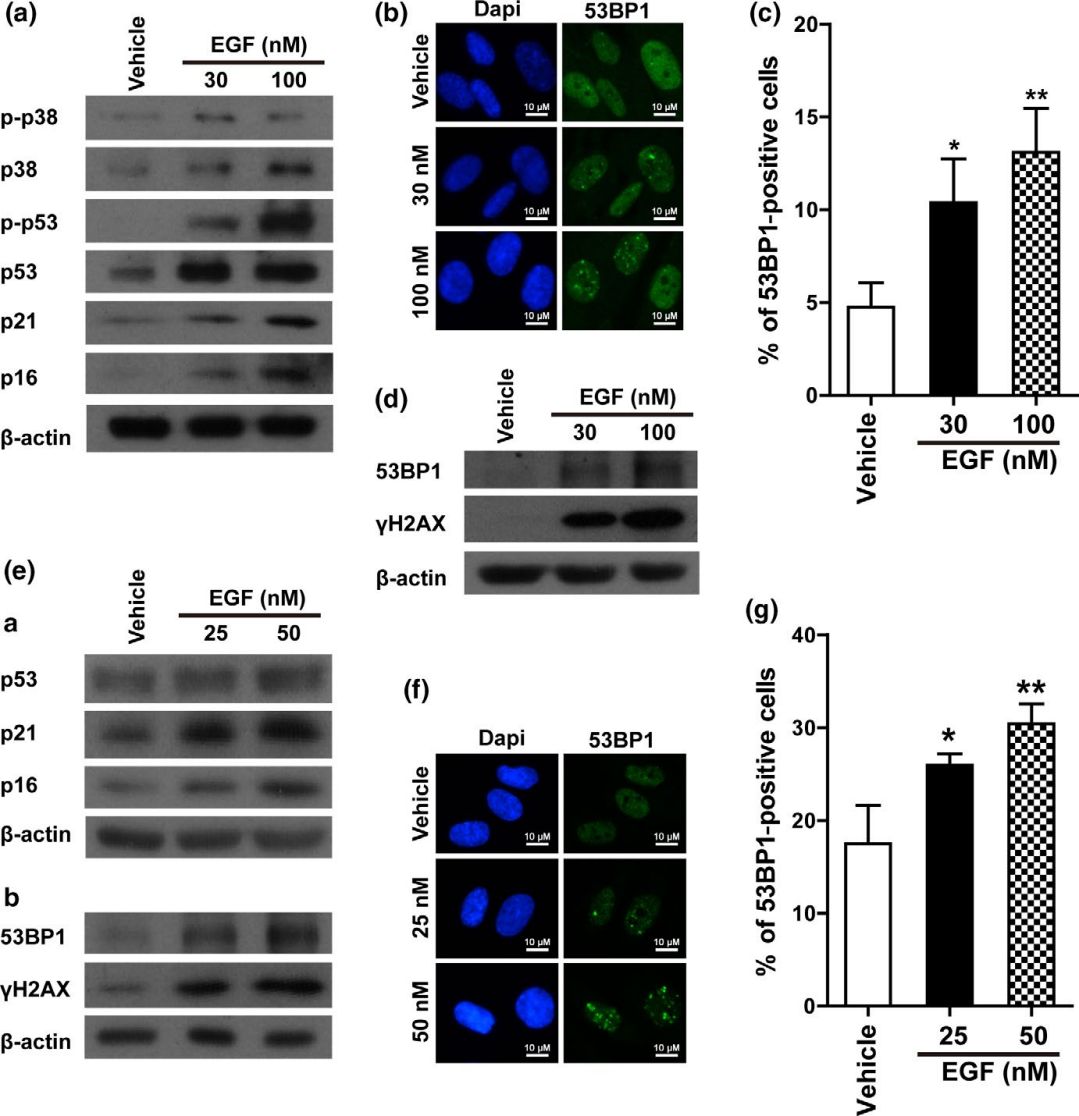
EGF treatment induced the activation of signaling pathways involved in Ras‐induced senescence. (a) Protein levels of p‐p38 (Tyr182), p38, p‐p53 (Ser15), p53, p21, and p16 in IMR90 cells with or without EGF treatment at the indicated concentrations for 3 days. (b and c) Representative pictures of immunofluorescence staining of 53BP1 and statistical analysis of the positive cells in IMR90 cells with or without EGF treatment at the indicated concentrations for 3 days. (d) Protein levels of 53BP1 and γH2AX in above cells. (e) a, Protein levels of p53, p21, and p16 in HUVECs with or without EGF treatment at the indicated concentrations for 3 days; b, protein levels of 53BP1 and γH2AX in above cells. (f and g) Representative pictures of immunofluorescence staining of 53BP1 and statistical analysis of the positive cells in HUVECs with or without EGF treatment at the indicated concentrations for 3 days. Data indicate the mean values calculated from three independent experiments (±*SD*). The Western blots were performed at least three times, and the representative pictures are presented

Next, we investigated the molecular mechanisms through which EGF induces senescence. Expression levels of proteins involved in RIS, namely p‐p38, p38, p‐p53, p53, p21, and p16, were notably upregulated in IMR90 cells after EGF treatment (Figure 4a). RIS is often accompanied by DNA damage accumulation, thus we also examined the formation of 53BP1 foci and protein levels of 53BP1 and γH2AX in IMR90 cells after EGF treatment using immunofluorescence and Western blotting. As shown in Figure 4b‐d, increased 53BP1 foci formation and protein levels of 53BP1 and γH2AX indicated DNA damage accumulation in IMR90 cells treated with EGF. In addition, when HUVECs were treated with different concentrations of EGF, the protein levels of p53, p21, and p16 were also upregulated (Figure 4e‐a). Similar to IMR90 cells, more DNA damage was accumulated in HUVECs after EGF treatment, as manifested by upregulated protein levels of 53BP1 and γH2AX (Figure 4e‐b) and significantly increased 53BP1 foci formation (Figure 4f,g). These data together suggest that EGF treatment induces cellular senescence in IMR90 cells and HUVECs via the activation of signaling pathways involved in RIS.

### 2.5 EGF treatment induces cellular senescence through activation of the Ras‐BRaf pathway

**FIGURE 5:**
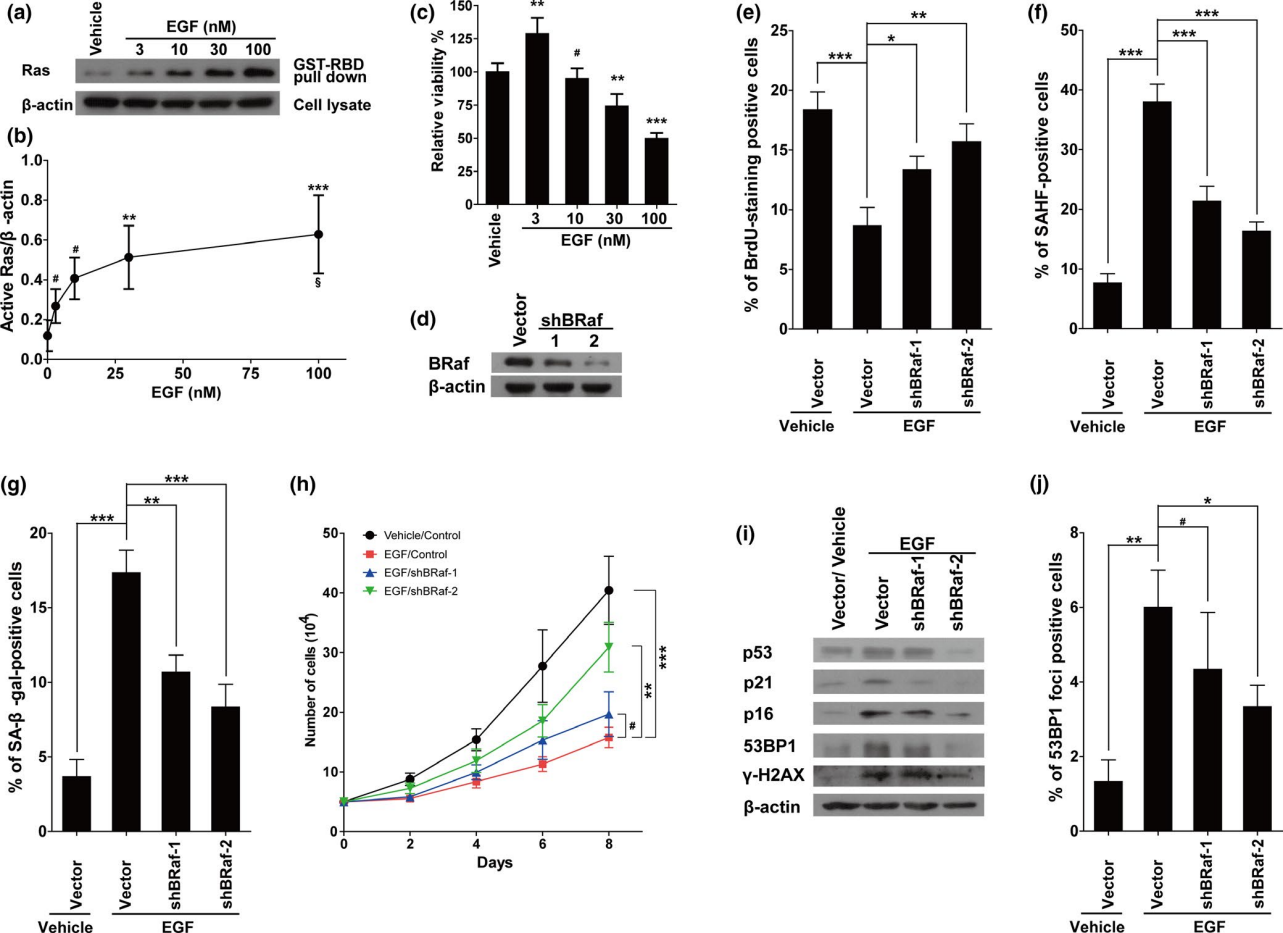
EGF‐induced cellular senescence is dependent on Ras‐BRaf activation. (a) IMR90 cells were treated with EGF at the indicated concentrations for 10 min before GST‐Raf‐RBD pull‐down assays. Levels of GTP‐bound Ras were determined. The experiments were repeated for four times, and blots were quantified using NIH ImageJ software. The amount of GTP‐bound Ras was normalized to β‐actin of the same group. (b) The statistical analysis of a. (c) The viabilities of the cells treated with EGF at different concentrations for 3 days. (d) Protein levels of BRaf in IMR90 cells with or without BRaf knockdown. (e–g), Statistical analysis of positive cells from BrdU (e), SAHF (f), or SA‐β‐gal staining (g) in the groups indicated in the figures. (h) The growth curves of the cells with indicated treatments. (i) Protein levels of p53, p21, p16, 53BP1, and γH2AX in above cells. (j) Statistical analysis of 53BP1 foci‐positive cells in the groups indicated in the figures. Data indicate the mean values calculated from three independent experiments (±*SD*). The Western blots were performed at least three times, and the representative pictures are presented. **p < .01; ***p < .001; #*p* > .05; compared to the vehicle group. §*p* < .05; compared to the 3 nM group

To further confirm our hypothesis, we examined the expression of active Ras in EGF‐treated cells using the Active Ras Pull‐Down and Detection Kit. As shown in Figure 5a,b, the protein levels of active Ras increased as the concentration of EGF applied to the cells increased. The levels of active Ras in the 30 and 100 nM groups were significantly upregulated compared with the vehicle group. In addition, there was more active Ras in the 100 nM group than in the 3 nM group. Similarly, EGF treatment increased active Ras in HUVECs (Figure S8). Since the activation of EGFR and Ras signaling pathways is well known to promote cell proliferation, we next examined the viability of the cells treated with different concentrations of EGF for 3 days. As shown in Figure 5c, while cell proliferation was mildly (but significantly) increased with 3 nM of EGF, cell proliferation was significantly inhibited with 30 or 100 nM of EGF. These results show that as concentration increases, the role of EGF changes from promoting to inhibiting proliferation of IMR90 cells.

Ras activation often induces cellular senescence through activation of Ras‐BRaf‐Erk signaling cascades, thus we next engineered BRaf knockdown in IMR90 cells (Figure 5d), and found marked inhibition of EGF‐induced cellular senescence (Figure 5e–h), as manifested by increased percentages of BrdU‐positive cells, decreased percentages of SAHF‐ or β‐gal‐positive cells, and improved cell proliferation. In addition, BRaf knockdown inhibited the upregulation of molecules involved in RIS, including p16, p21, and p53, as well as DNA damage accumulation manifested by decreased protein levels of 53BP1 and γH2AX and decreased numbers of 53BP1 foci (Figure 5i,j).

### 2.6 Erk inhibition impedes cellular senescence induced by EGF treatment

**FIGURE 6:**
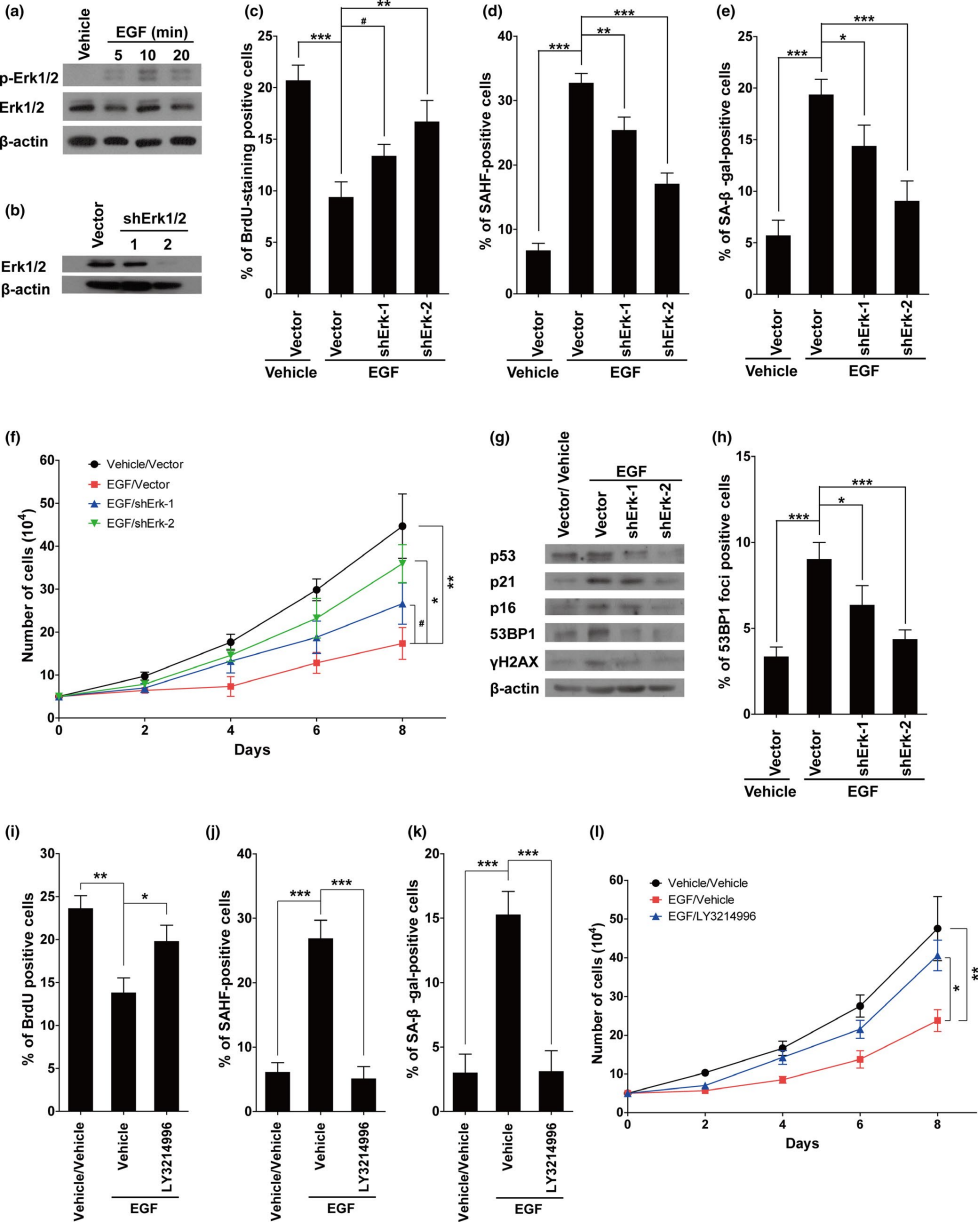
Erk1/2 activation is necessary for EGF‐induced cellular senescence. (a) Cells were treated with or without 100 nM of EGF (for 5, 10, or 20 min), and the protein levels of p‐Erk1/2 and Erk1/2 were determined by Western blotting. (b) Protein levels of Erk1/2 in cells with or without Erk1/2 knockdown. (c–e) Statistical analysis of positive cells from BrdU (c), SAHF (d), or SA‐β‐gal staining (e) in the groups indicated in the figures. (f) the growth curves of the cells with indicated treatments. (g) Protein levels of p53, p21, p16, 53BP1, and γH2AX in above cells. (h) The statistical analysis of 53BP1 foci‐positive cells in the groups indicated in the figures. (i–k), Statistical analysis of positive cells from BrdU (i), SAHF (j), or SA‐β‐gal staining (k) in the groups indicated in the figures; LY3214996 (300 nM), a selective Erk1/2 inhibitor, was applied together with EGF (100 nM). (l), The growth curves of the cells with indicated treatments. Data indicate the mean values calculated from three independent experiments (±*SD*). The Western blots were performed at least three times, and the representative pictures are presented

Phosphorylation of Erk1/2 (p‐Erk1/2, active form of Erk1/2) is a typical downstream indicator of EGFR or Ras activation, and as expected, the expression of p‐Erk1/2 was notably upregulated in EGF‐treated cells (Figure 6a). Similar to BRaf knockdown, Erk1/2 knockdown in the IMR90 cells (Figure 6b) also significantly inhibited EGF‐induced cellular senescence (Figure 6c–f), as manifested by increased DNA synthesis, decreased SAHF‐ and β‐gal‐positive cells, and improved cell proliferation. Similarly, protein levels of p16, p21, p53, 53BP1, and γH2AX were decreased in Erk1/2 knockdown cells (Figure 6g). In addition, numbers of 53BP1 foci‐positive cells decreased significantly in Erk1/2 knockdown cells (Figure 6h). In addition, we chose LY3214996, a selective and novel Erk1/2 inhibitor, to further test our hypothesis. As expected, LY3214996 treatment dramatically inhibited cellular senescence induced by EGF treatment, as manifested by increased DNA synthesis, decreased SAHF‐ and β‐gal‐positive cells, and improved cell proliferation (Figure 6i–l). The above results illustrate that EGF induces cellular senescence through Erk activation.

### 2.7 MCP‐3 may induce cellular senescence through a pathway independent of EGFR activation

Our earlier results showed that inhibition of the EGFR pathway had no effect on MCP‐3‐induced cellular senescence. We next compared the effects of the senescence‐inducing cytokines in the presence of EGF treatment. As shown in Figure S9, only MCP‐3 treatment significantly increased cellular senescence on the basis of EGF stimulation, suggesting that MCP‐3 can function in an EGFR‐independent pathway.

### 2.8 EGFR activation induces secretion of IL‐8 and matrix metalloproteinase (MMP)‐3 from IMR90 cells

It would be interesting to determine whether activation of EGFR is able to further amplify SASP. To answer this question, we first examined the mRNA levels of several typical SASP factors. The results showed that the mRNA levels of IL‐8 and MMP‐3 were significantly upregulated by 100 nM of EGF treatment (Figure S10A). We next used enzyme‐linked immunosorbent assay (ELISA) kits to measure the levels of IL‐8 and MMP‐3 in conditioned media of the cells with different treatment. As shown in Figure S10B, treatment with 100 nM of EGF significantly increased the secretory levels of IL‐8 and MMP‐3. Then, we used various factors (each at its optimal concentration) to stimulate cells. As expected, the levels of IL‐8 and MMP‐3 were significantly increased after cytokine stimulation (Figure S10C,D). Of note, there was no significant difference among the levels of IL‐8 and MMP‐3 induced by the other factors, except for the secretory levels induced by IL‐1β, which were significantly higher than with the other factors. These results suggest that in addition to activating EGFR, IL‐1β may induce IMR90 cells to secrete IL‐8 and MMP‐3 through other pathways.

### 2.9 EGFR activation cannot induce senescence in cancer cells or quiescent cells

EGFR activation is well known to promote survival of certain cancer cells. It is interesting to determine whether EGFR activation can induce cellular senescence in cancer cells. To address this question, we measured the viabilities of cancer cells, MGC‐803 and PC‐9, after they were treated with different cytokines or EGF for 3 days. As shown in Figures S11A–E and S12A–E, none of the cytokines significantly inhibited proliferation of either MGC‐803 or PC‐9 cells. Therefore, we speculated that these cytokines potentially do not induce cellular senescence in these cancer cells. EGF treatment also did not inhibit cell proliferation in the cancer cells (Figures S11F and S12F). The results of SAHF (Figures S11G and S12G) and β‐gal staining (Figures S11H and S12H) confirmed that EGF treatment could not induce cellular senescence in these cancer cells.

Since most cells in the body rest in a quiescent state, we were also interested in determining whether these senescence‐inducing cytokines are also capable of inducing senescence in quiescent cells. We first established quiescent IMR90 cells by contact inhibition and low fetal bovine serum (FBS; 0.5%), and then treated the quiescent IMR90 cells with EGF and the cytokines. None of these treatments increased SAHF‐positive cells (Figure S13). We did not use the β‐gal staining method in this experiment as β‐gal staining may give false positive results when the cells are too crowded. Overall, the above results suggest that EGFR activation cannot induce senescence in quiescent cells.

## 3 DISCUSSION

Aging gracefully is the ultimate dream of all mankind, especially when many countries are in the quagmire of population aging. Scholars believe that inflammation plays a causal role in aging, supported by the results of an important longitudinal study of semi‐supercentenarians (Arai et al., 2015). More specifically, several pro‐inflammatory cytokines, including IL‐1β, IL‐6, and TGF‐β1, have been extensively studied due to their roles in the aging. On the other hand, senescent cells can promote organism aging. In this study, we systematically assessed 21 pro‐inflammatory cytokines and found that 6 of them were able to induce cellular senescence in both IMR90 cells and HUVECs. The subsequent mechanism studies show that 5 out of 6 senescence‐inducing cytokines induce cellular senescence through activation of the EGFR‐RAS signaling pathway.

As a member of the ErbB/HER family, EGFR is widely expressed on the cell membrane in many different tissues. After activation, EGFR‐mediated signaling pathways exert profound effects on physiological functions of cells, such as survival, proliferation, differentiation, and migration. Surprisingly then, very few articles have discussed the roles of EGFR‐mediated signaling pathways in cellular senescence, and those that did demonstrated a suppressive role for EGFR signaling on cellular senescence (Liu et al., 2015). For example, Alexander et al. (2015) reported that EGF promotes mammalian cell growth by suppressing cellular senescence. Yao et al. (2015) found that EGF protects cells against Dox‐induced growth arrest through activating cyclin D1 expression. On the clinical front, Wang et al. (2011) reported that inhibiting EGFR radiosensitizes non‐small‐cell lung carcinoma cells by inducing senescence through sustaining DNA double‐strand breaks. In contrast, our present study indicated that active EGFR signaling might induce cellular senescence via activation of Ras. In support of these findings, Yaglom's group showed that expression of NueT (a mutant‐activated rodent isoform of Her2) induces cellular senescence (either fully or partially) in human mammary epithelial cells (Sherman, Meng, Stampfer, Gabai, & Yaglom, 2011). While slight to mild activation of Ras promotes cell growth, over‐activated Ras signaling usually induces cellular senescence (Deng, Liao, Wu, & Sun, 2004). Our results now suggest that certain pro‐inflammatory cytokines can induce cellular senescence through activating EGFR, which in turn over‐activates Ras signaling. Moreover, the activation of EGFR and Ras signaling pathways was previously well known to promote cell proliferation and survival of certain tumors, so we further clarified that neither EGF nor cytokines induced senescence in cancer cell lines under our experimental conditions (Figures S11 and S12). Considering that many cancer cell lines lack functional signaling pathways to establish cellular senescence (the MGC‐803 and PC‐9 cells used herein contain mutant p53), we believe our findings are more relevant to normal physiological aging.

It is well recognized that during wound healing, stationary fibroblasts distant from the wound area are activated and migrate to the wound site, where they synthesize extracellular matrix proteins, such as collagen, to help close wounds. Inflammatory factors play roles in the activation and chemotaxis of fibroblasts. In addition, recent studies have revealed the importance of cellular senescence in wound healing (Baker et al., 2016; Demaria et al., 2014). Our results show that inflammatory factors induce senescence in proliferating but not quiescent fibroblasts (Figure 3 and Figure S13). Such increasing evidence implies that cellular senescence of fibroblasts induced by certain inflammatory factors might play a role in wound healing.

We further showed in this paper that EGFR activation induces secretion of certain major inflammatory factors. Consistent with our results, recent studies demonstrated that activating the EGFR signaling pathway could activate inflammatory responses in certain physiological or pathological processes (Zaiss, Gause, Osborne, & Artis, 2015). More specifically, studies revealed a crosstalk between EGFR and NF‐κB signaling pathways (Shostak & Chariot, 2015; Tanaka et al., 2011). Furthermore, Alberti et al. (2012) showed that EGF treatment increased the production of IL‐6 and IL‐8 in epithelial ovarian cancer cells. As mentioned before, activation of NF‐κB plays a central role in the production of secretory factors during cellular senescence. Now, our results imply that a positive feedback loop consisting of EGFR, Ras, NFκB, and SASP factors might exert a potential, but substantial function during the processes of tissue aging.

Overall, our study offers important insight into a novel and long‐ignored mechanism through which EGFR regulates cellular senescence. While more evidence is definitely needed to fully characterize the pathways, our results imply that growth signals themselves may catalyze aging.

## 4 EXPERIMENTAL PROCEDURES

### 4.1 Antibodies and other reagents

The antibodies used in the current study are listed in Table S1. The other reagents used are as follows: recombinant EGF, IL‐1β, IL‐7, IL‐13, IL‐15, GRO‐α, IL‐8, SDF‐1α, MCP‐3, MIP‐1α, MCP‐2, MIP‐3α, eotaxin‐3, bFGF, KGF, vEGF, IGF‐BP7, OPG, GM‐CSF (BBI Life Sciences, Shanghai, China); recombinant IL‐6, MMP‐3, TGF‐β1 (PeproTech); cetuximab (Merck KGaA); gefitinib, cisplatin, and LY3214996 (Selleck Chemicals Inc); MTT (3‐[4]‐2,5‐ diphenyltetrazolium bromide thiazolyl blue), X‐Gal (5‐bromo‐4‐chloro‐3‐indolyl‐β‐D‐galactopyranoside), DAPI (4′, 6‐diamidine‐2′‐phenylindole dihydrochloride), BrdU (5‐bromo‐2‐deoxyuridine), and PI (propidium iodide) (Sigma‐Aldrich); annexin V‐FITC/PI Apoptosis Detection Kit (Yeasen Biotech); Human IL‐8 Uncoated ELISA Kit (Thermo Scientific); Human Total MMP3 ELISA Kit and Human EGF ELISA Kit (Proteintech).

### 4.2 Primary culture of HUVECs

HUVECs were isolated and cultured as previously described (Poon, Zhang, Dunsky, Taubman, & Harpel, 1997). Briefly, fresh umbilical cord was washed three times with sterile PBS and placed in a 10‐cm plate. The venous vessel was then digested with 0.1% collagenase IV (Sigma) for 20 min at 37°C, and the collected cells were cultured in EGM™‐2 BulletKit™ media (LONZA) with penicillin/streptomycin (Life Technologies). The cultured HUVECs (PD 4‐8) were used in the following experiments, and any unused cells were placed in liquid nitrogen for cryopreservation. This study was approved by the Human Research Ethics Committee of Jiangsu University. All patients provided signed informed consent.

### 4.3 Cell culture and drug treatment

IMR90 cells, a generous gift from Rugang Zhang's Lab at Wistar Institute, were cultured in DMEM, supplemented with 10% FBS, vitamin solution, amino acids, non‐essential amino acids, 2 mM sodium pyruvate, penicillin, and streptomycin (Life Technologies). 293FT cells were cultured in DMEM, supplemented with 10% FBS, non‐essential amino acids, 2 mM sodium pyruvate, penicillin, and streptomycin (Life Technologies). In order to avoid cellular senescence caused by hyperoxia, a low oxygen incubator (2% O_2_, Thermo Fisher Scientific, model # 4131) was used. The cell passages used in this study were 25–35.

For some experiments, IMR90 cells were made quiescent by first culturing them to confluent and then starving them in media containing 0.5% FBS for 2 days.

Cells (3 × 10^4^) were plated in a 24‐well flat‐bottomed plate and cultured with medium containing various concentrations of cytokine candidates for two days. Then, treated cells were used for DAPI and SA‐β‐gal staining. Cetuximab (10 μg/ml) and gefitinib (100 ng/ml) were applied together with the cytokines.

PC9 and MGC‐803 cells were cultured in RPMI 1640 and DMEM, respectively, supplemented with 10% FBS, penicillin, and streptomycin. The cells were plated in a 96‐well flat‐bottomed plate at 2 × 10^3^ cells/well and cultured with medium containing various concentrations of cytokines for two days. Then, cell viability was determined using the MTT assay.

### 4.4 Lentivirus infections

Lentivirus‐encoded short hairpin RNAs were purchased from BGI (Shenzheng, Guangdong, China), with sequences listed in Table S2. Production and infection of lentivirus were performed as described previously (Zhang et al., 2005). Briefly, lentivirus was packaged using a Virapower kit from Invitrogen by following the manufacturer's instructions. Cells infected with viruses encoding resistance to puromycin were selected in 1 μg/ml puromycin.

### 4.5 Growth curves

Cells (5 × 10^4^) were plated in a 3.5‐cm dish and cultured with medium containing various concentrations of EGF (3 dishes per group). Due to the low stability of EGF in culture condition (Masui, Castro, & Mendelsohn, 1993), fresh medium containing EGF was replaced every day. Cells were counted every 2 days. The experiment was independently repeated three times, and the representative results are presented.

### 4.6 Flow cytometry

To analyze the phase distribution and apoptotic status of the cells after appropriate treatments, the cells were collected and subjected to cell cycle and apoptosis assays. Cisplatin treatment (10 μM) and heat treatment (50°C for 10 min) were used in the cell apoptosis assay as the positive controls for methodology. The data were analyzed using FlowJo X software.

### 4.7 Immunofluorescence, SAHF staining, SA‐β‐gal staining, BrdU staining, and Western blotting

Treated cells were plated in 24‐well flat‐bottomed plates with coverslips and cultured for one day. Cells were then fixed in 4% paraformaldehyde solution for 10 min at room temperature, followed by immunofluorescence staining or SAHF staining using a method described in previous studies (Aird & Zhang, 2013). SA‐β‐gal, BrdU staining, and Western blotting were performed following the routine protocols in our laboratory. Images were captured using Nikon Eclipse.

### 4.8 Ras‐GTP assays

Cells were treated with 100 nM of EGF for 10 min and lysed in lysis buffer (25 mM Tris·HCl pH 7.2, 5 mM MgCl_2_, 150 mM NaCl, 1% NP‐40, and 5% glycerol) supplemented with 1 mM PMSF (Sangon Biotech) and 1% Halt Protease Inhibitor Cocktail EDTA‐Free (100×, Thermo Scientific). Next, protein concentrations were determined using the BCA (bicinchoninic acid) Protein Assay Kit (Beyotime Biotechnology) and GST‐Raf‐RBD pull‐downs were performed using the Active Ras Pull‐Down and Detection Kit (Thermo Scientific) (Young, Lou, & McCormick, 2013).

### 4.9 qRT‐PCR and ELISA assay

The qRT‐PCR experiments were carried out using the primers listed in Table S3. For ELISA assays, the levels of secreted IL8, MMP3, and EGF from conditioned medium were quantified with ELISA kits according to the manufacturer's instructions. The results were normalized against the average of the vehicle group in each assay.

### 4.10 Statistics

Data are presented as mean ± S.D. and were analyzed for significance between groups using Student's *t* tests (two‐tailed) or one‐way analysis of variance, unless otherwise noted. *p < *.05 was considered statistically significant. #*p* ≥ .05; *p < .05; **p < .01; ***p < .001.

## CONFLICT OF INTEREST

The authors have no conflicts of interest to declare.

## AUTHOR CONTRIBUTIONS

D Shang, D Sun, C Shi, and M Shen conducted the experiments. J Xu and X Hu analyzed and interpreted the data. H Liu and Z Tu conceived and designed the experiments. H Liu and Z Tu prepared the manuscript. All authors revised and agreed with the manuscript.

## Supporting information

Figure S1

Figure S2

Figure S3

Figure S4

Figure S5

Figure S6

Figure S7

Figure S8

Figure S9

Figure S10

Figure S11

Figure S12

Figure S13

Table S1

Table S2

Table S3

Table S4

Table S5

Table S6

Table S7

Table S8

Table S9

Table S10

Supplementary Material

## Data Availability

All original figures and data were deposited at https://pan.baidu.com/s/1-KLQDAD18bTG1-mO2KxiaQ with the password u22g.
