# Supplementary figures and images for: Activation of epidermal growth factor receptor signaling mediates cellular senescence induced by certain pro‐inflammatory cytokines
Source: Aging Cell. 2020 Apr 22;19(5):e13145. doi: 10.1111/acel.13145 (PMC7253070; doi:10.1111/acel.13145)

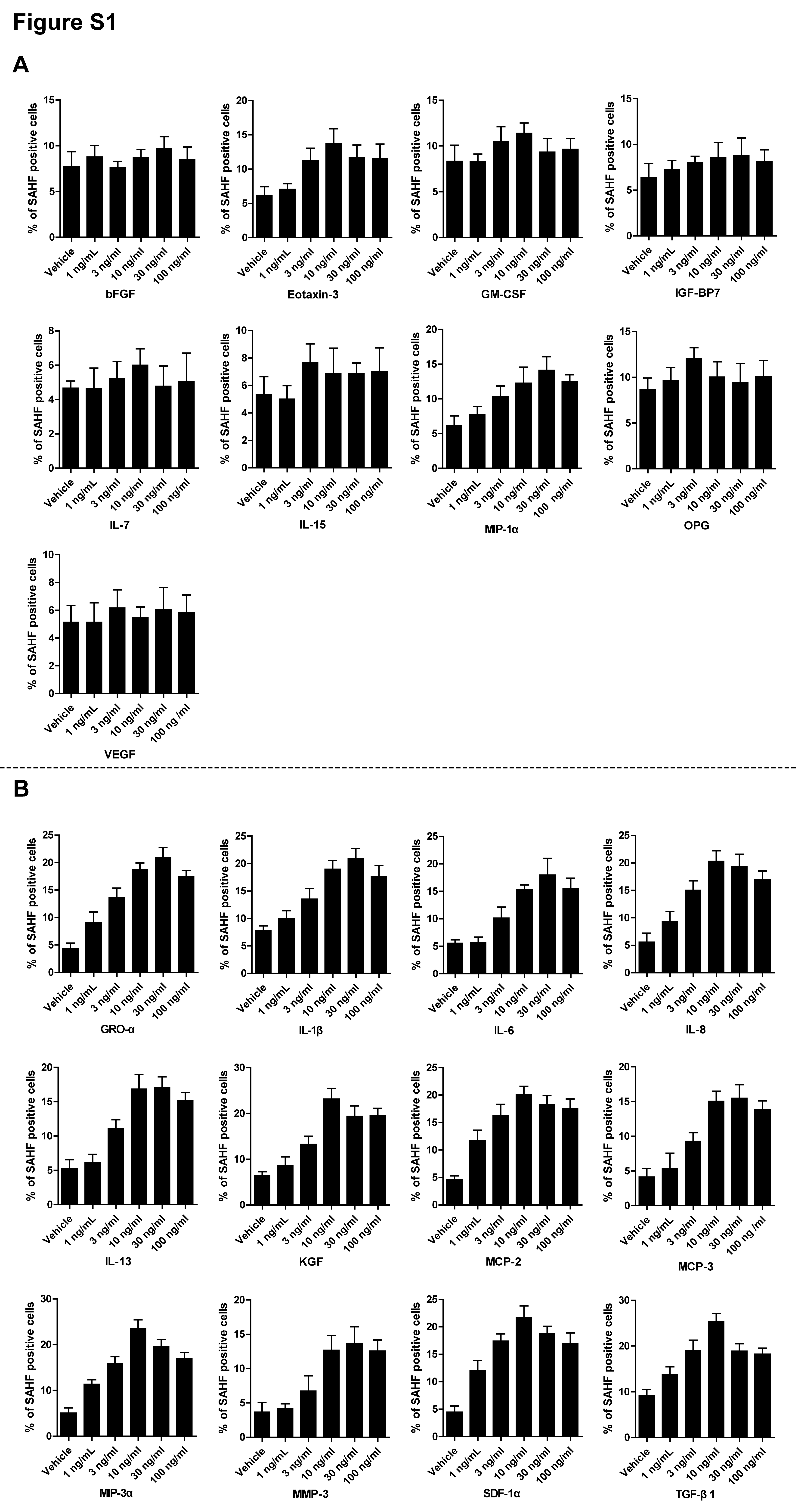

Supplement: Supplementary file 1 — Figure S1 [file ACEL-19-e13145-s001.tif]

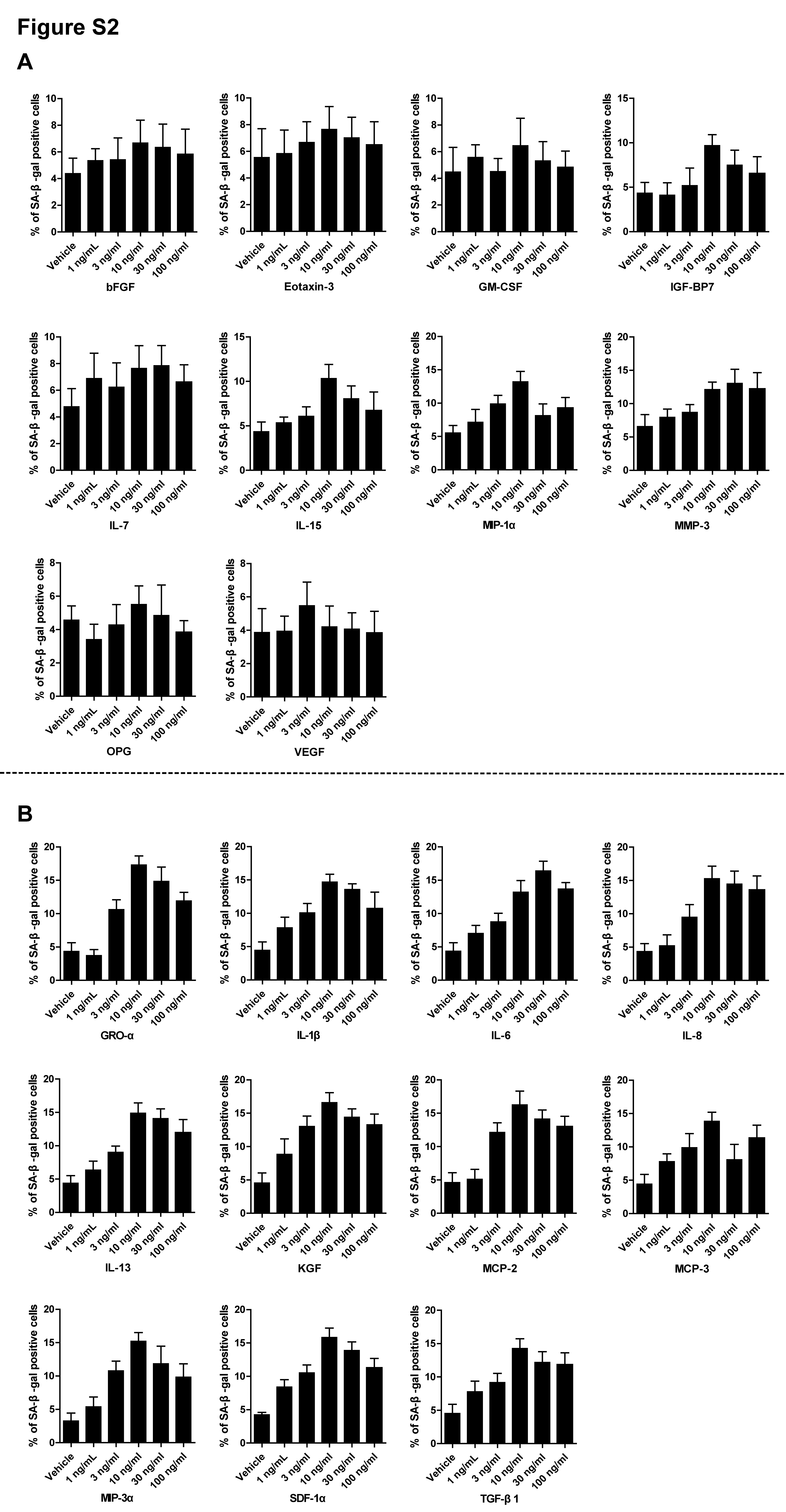

Supplement: Supplementary file 2 — Figure S2 [file ACEL-19-e13145-s002.tif]

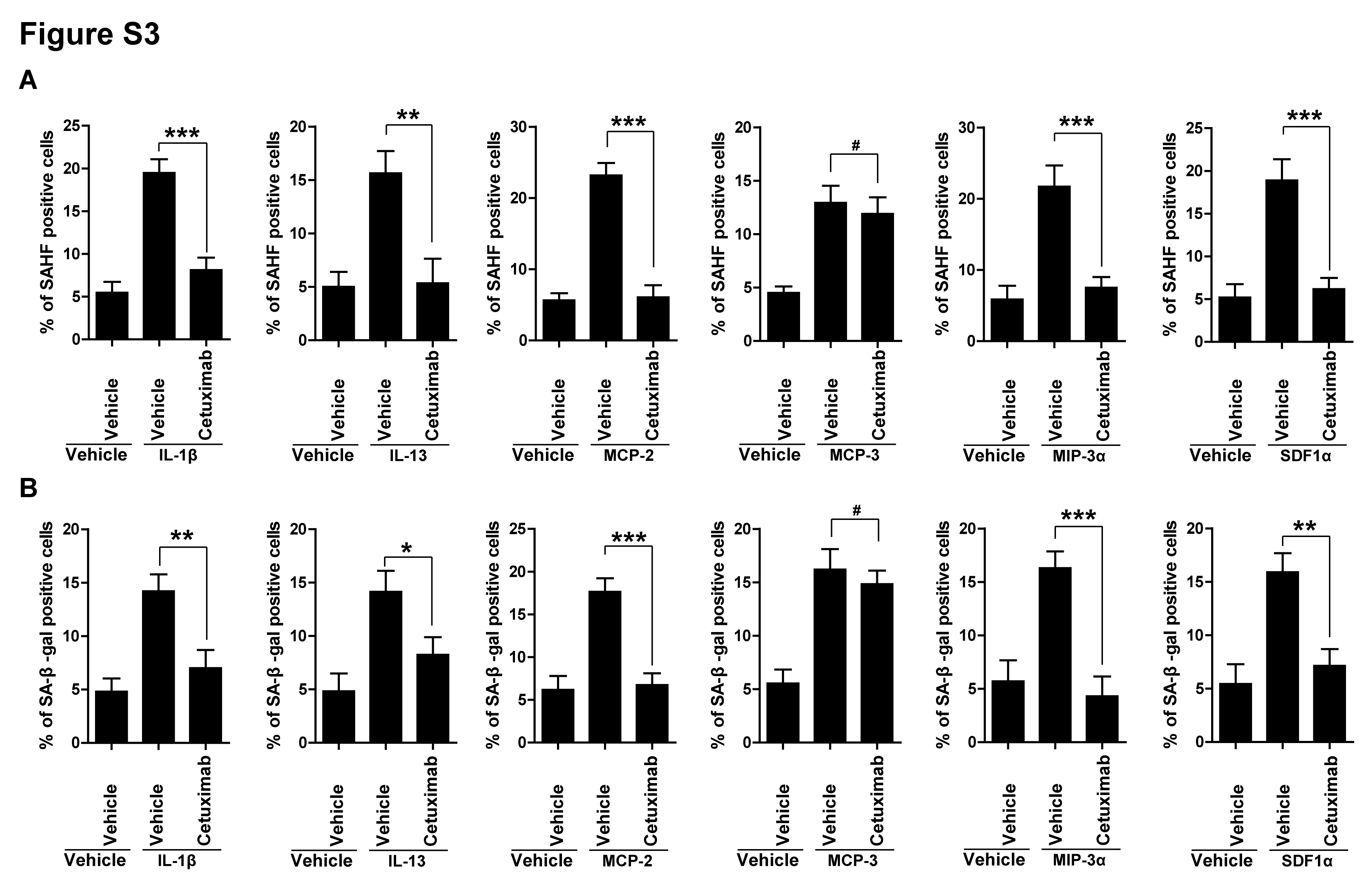

Supplement: Supplementary file 3 — Figure S3 [file ACEL-19-e13145-s003.tif]

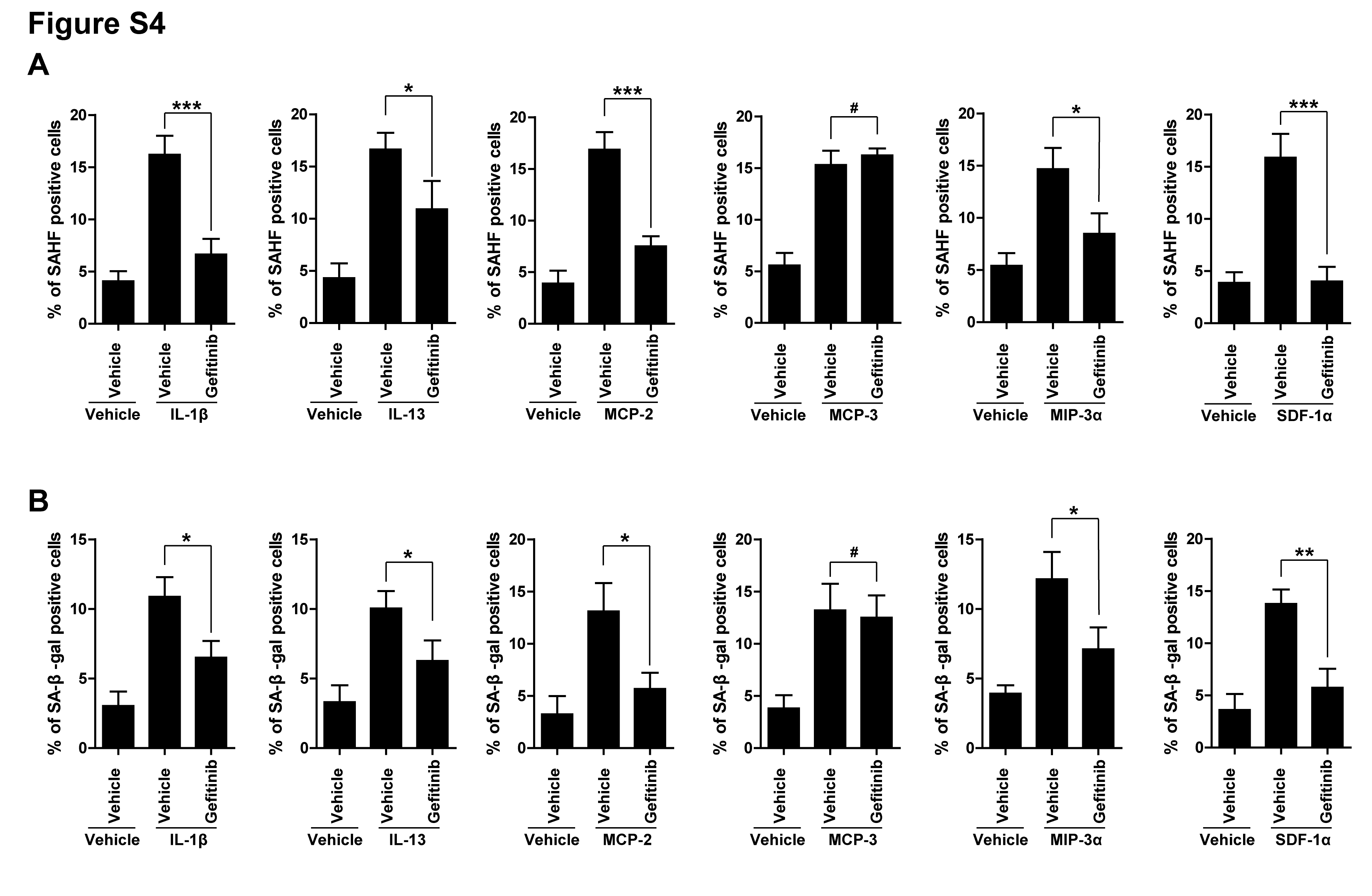

Supplement: Supplementary file 4 — Figure S4 [file ACEL-19-e13145-s004.tif]

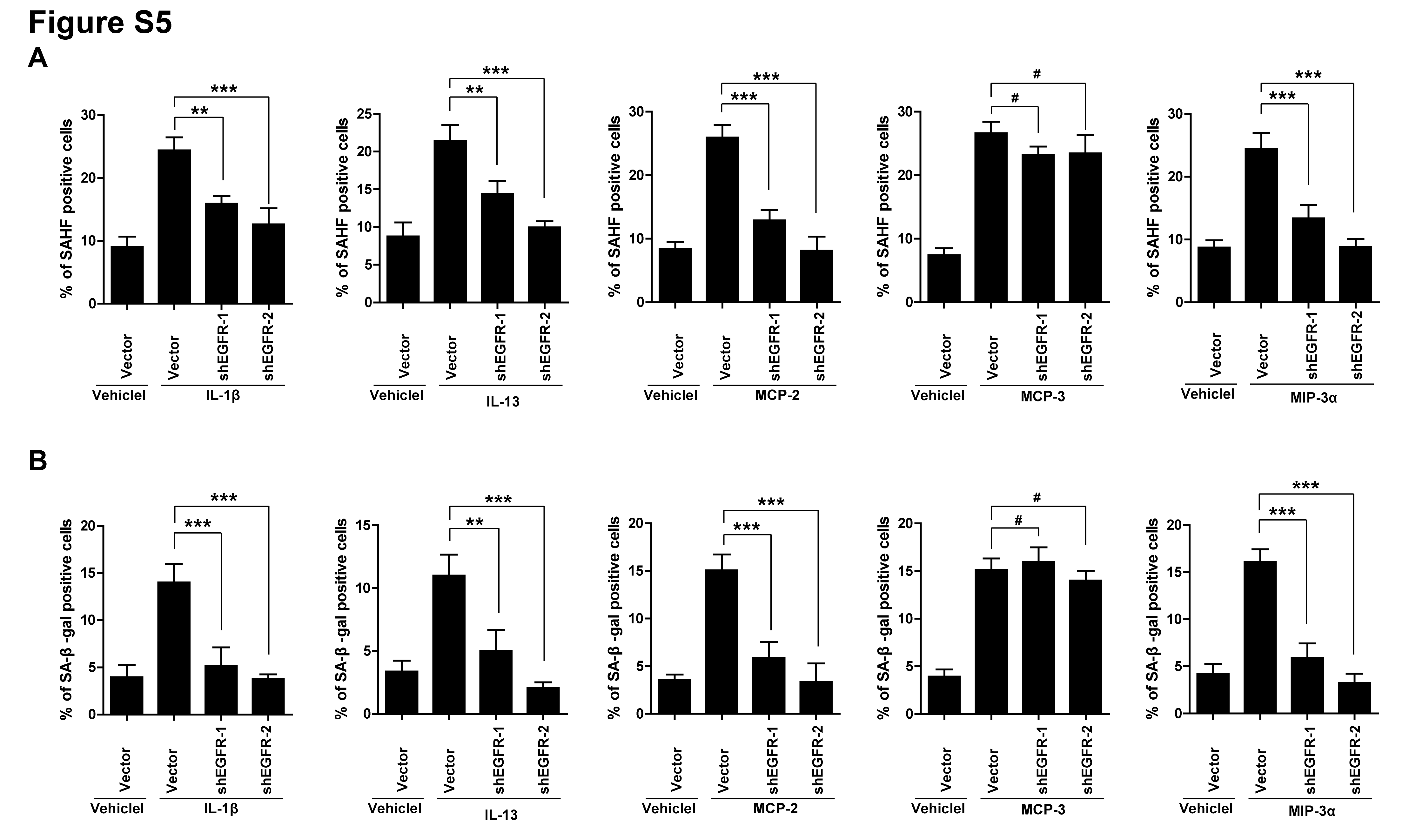

Supplement: Supplementary file 5 — Figure S5 [file ACEL-19-e13145-s005.tif]

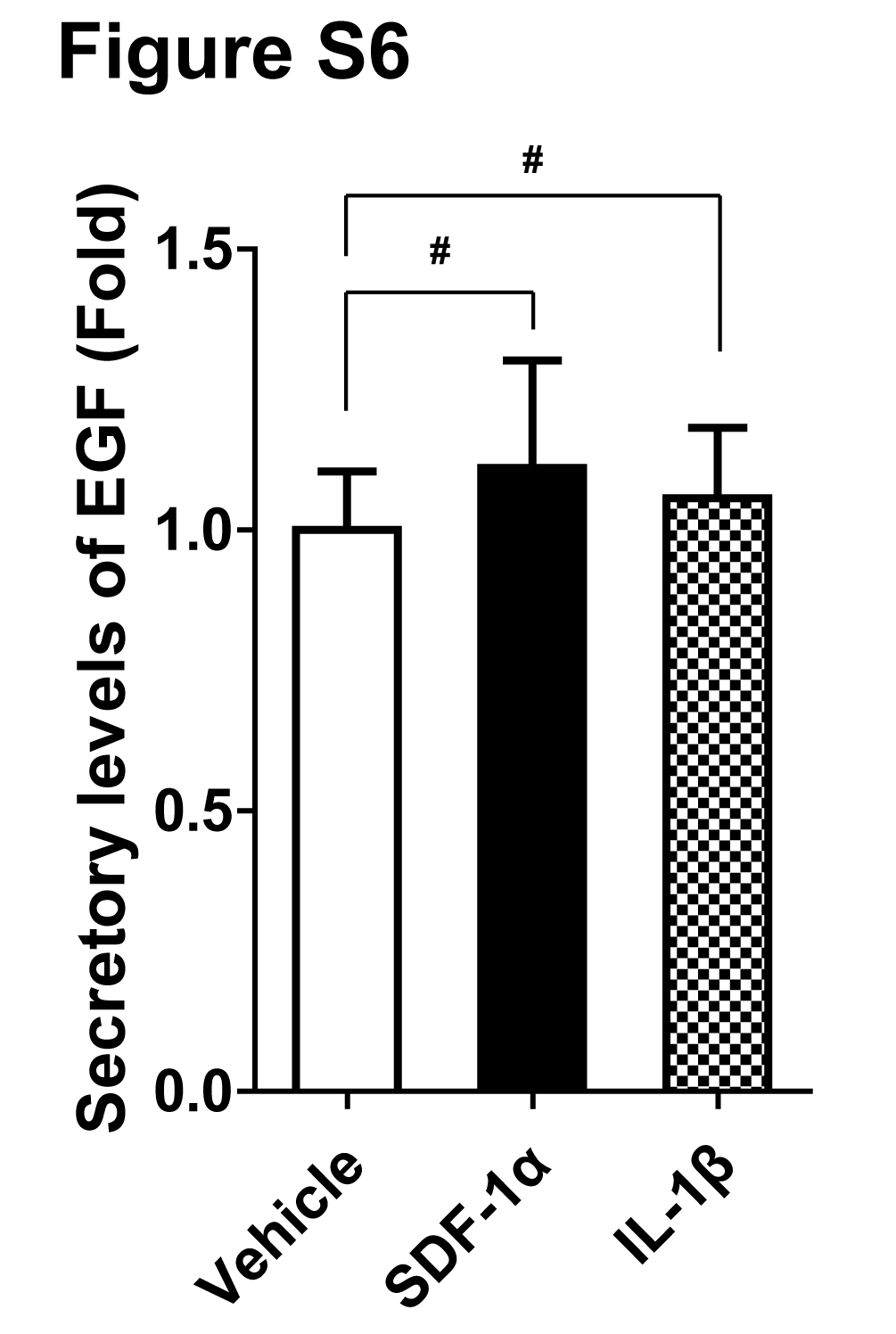

Supplement: Supplementary file 6 — Figure S6 [file ACEL-19-e13145-s006.tif]

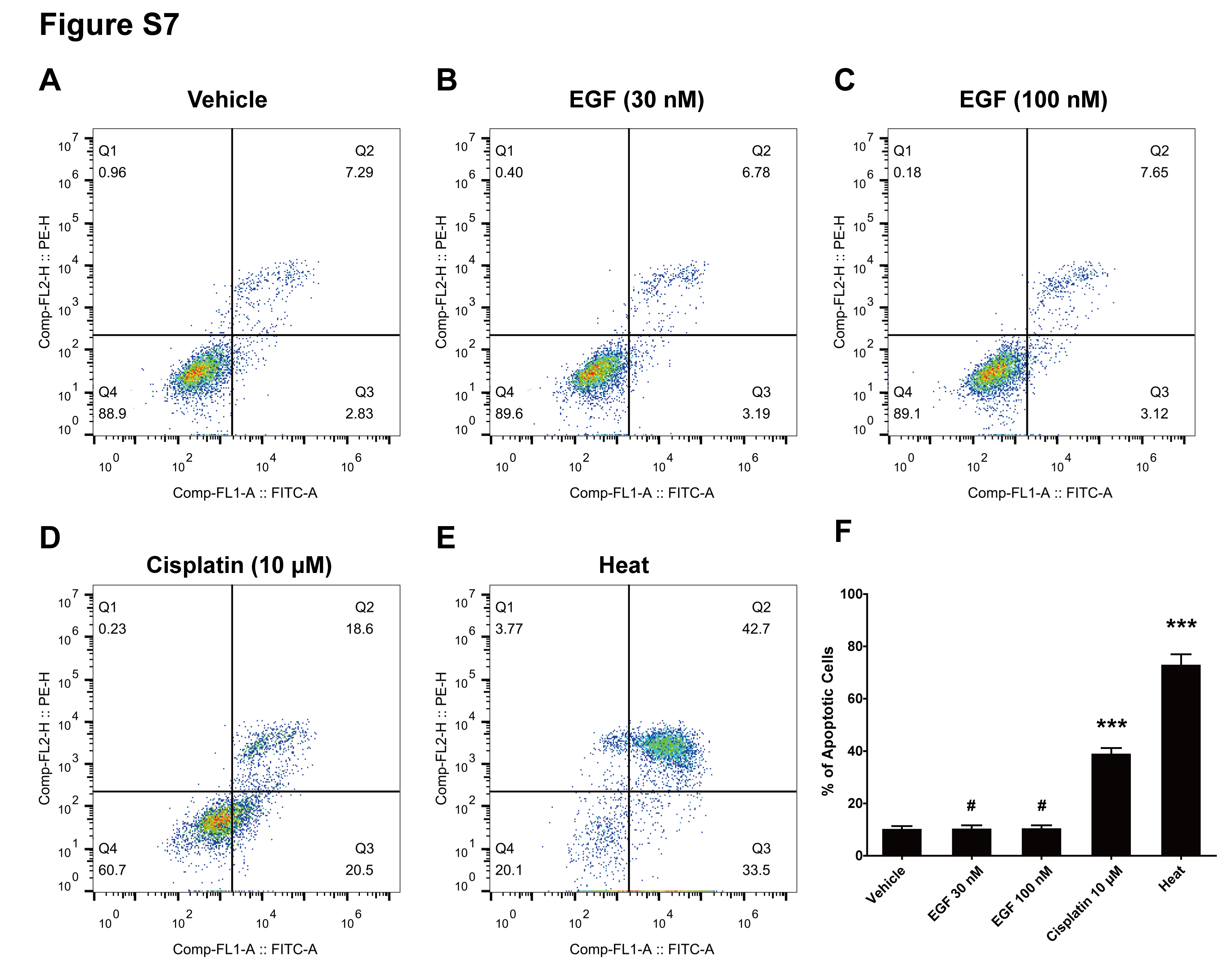

Supplement: Supplementary file 7 — Figure S7 [file ACEL-19-e13145-s007.tif]

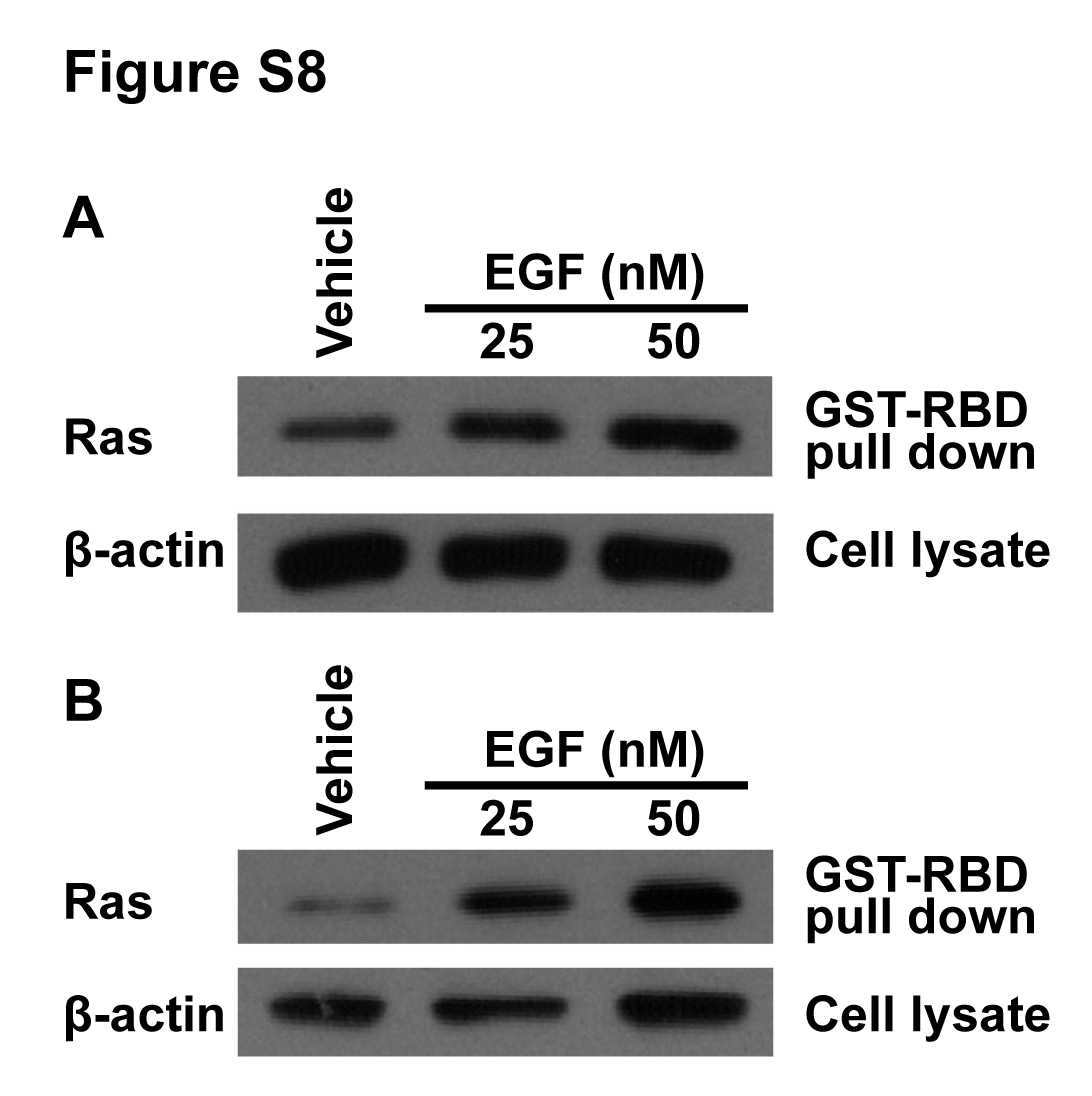

Supplement: Supplementary file 8 — Figure S8 [file ACEL-19-e13145-s008.tif]

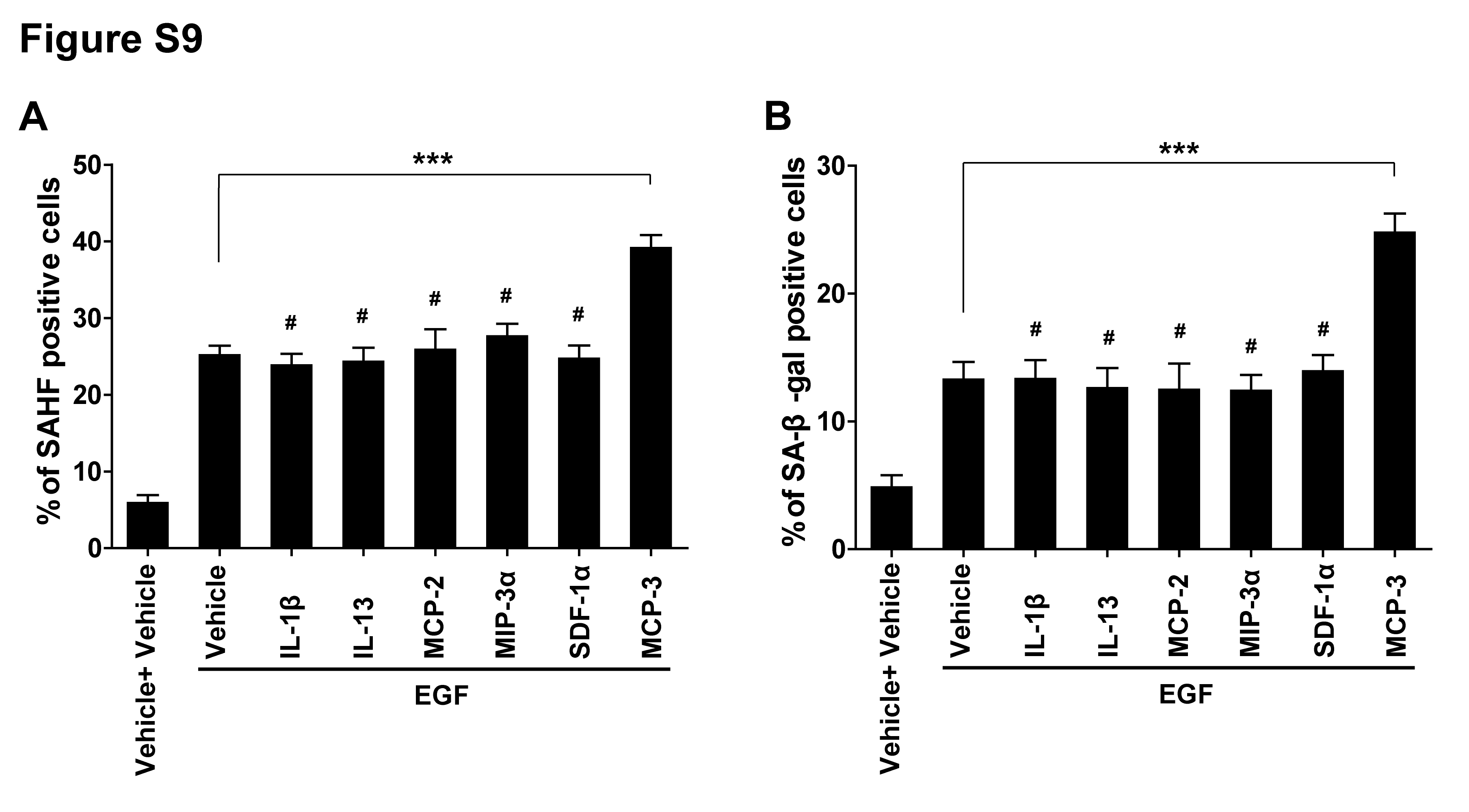

Supplement: Supplementary file 9 — Figure S9 [file ACEL-19-e13145-s009.tif]

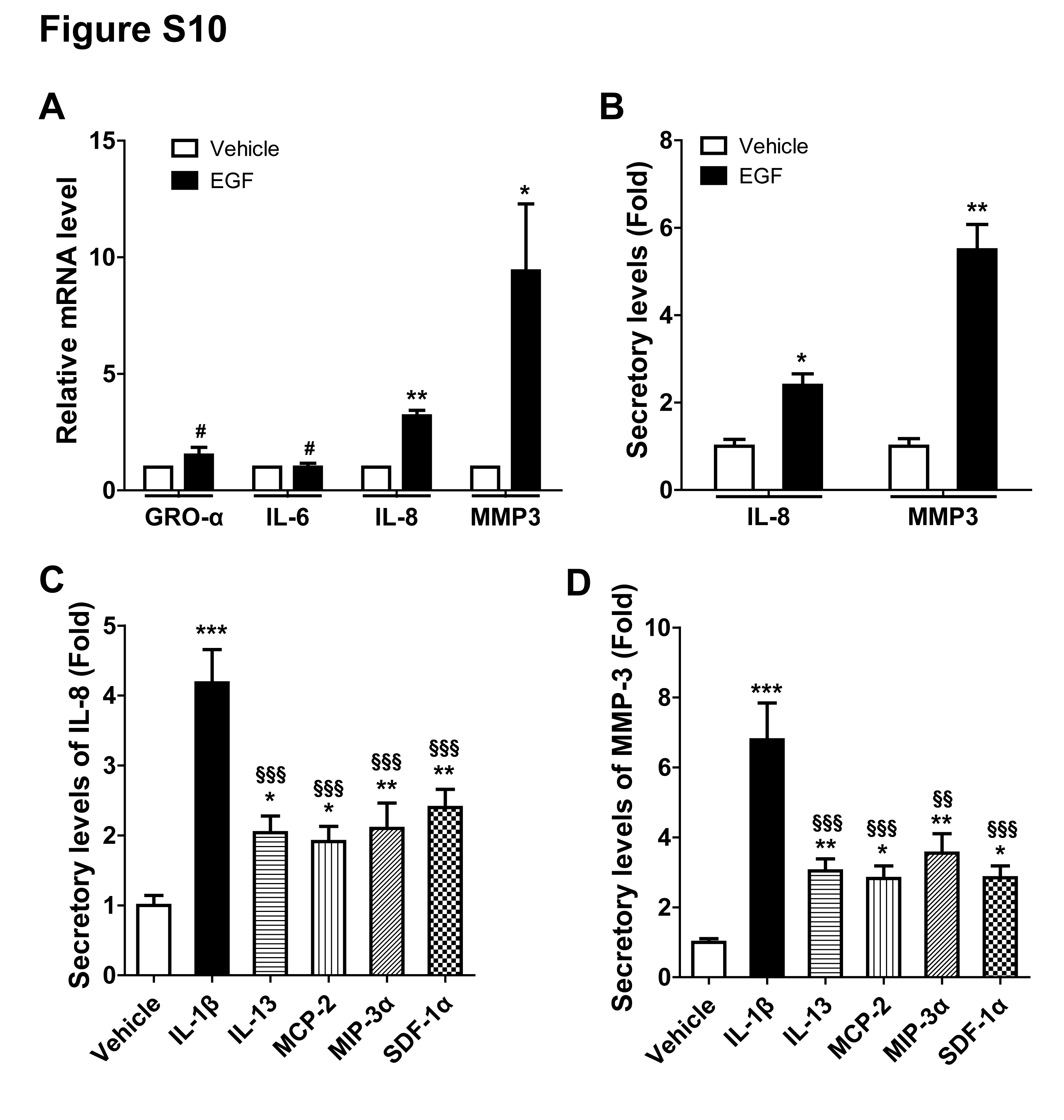

Supplement: Supplementary file 10 — Figure S10 [file ACEL-19-e13145-s010.tif]

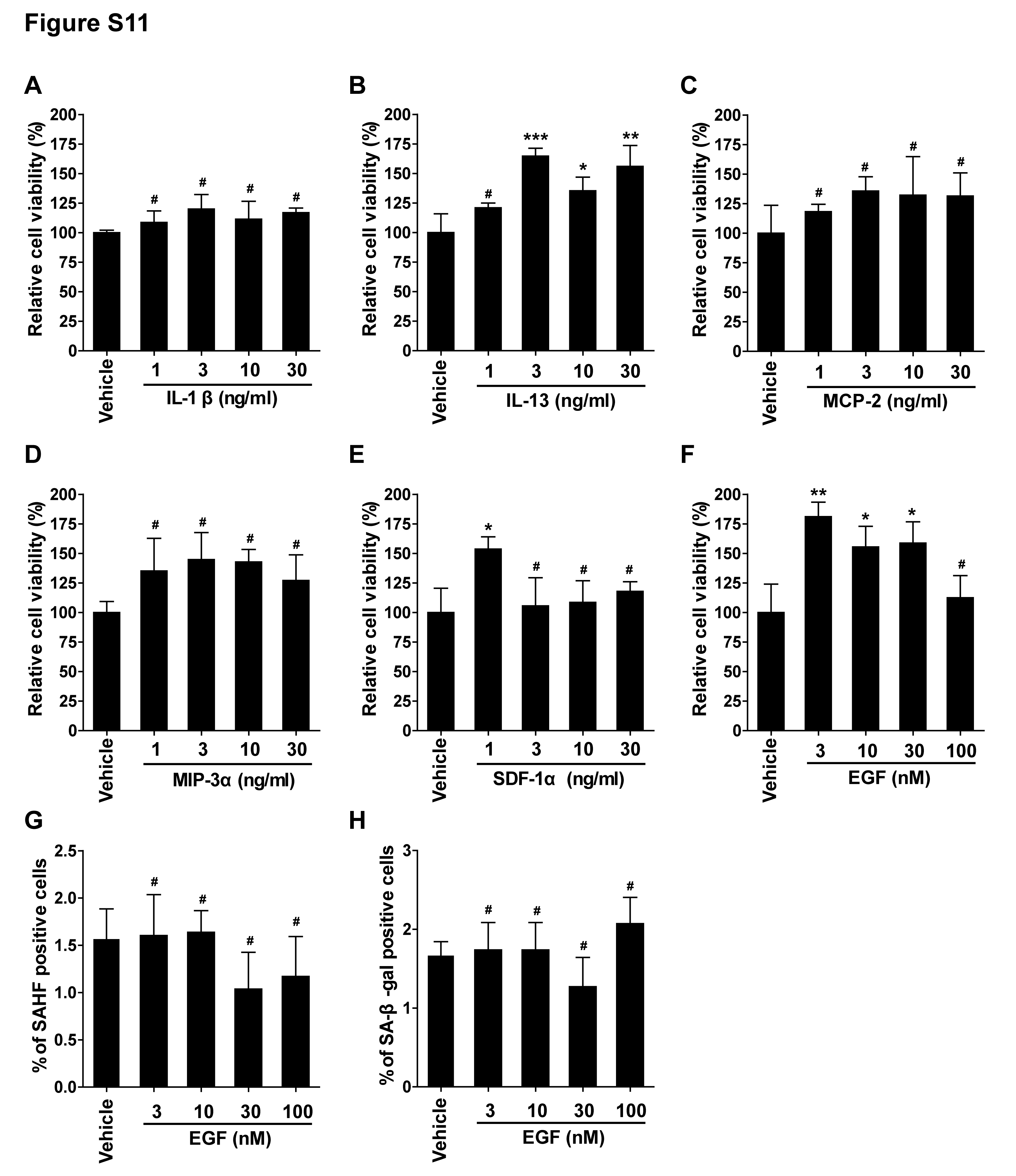

Supplement: Supplementary file 11 — Figure S11 [file ACEL-19-e13145-s011.tif]

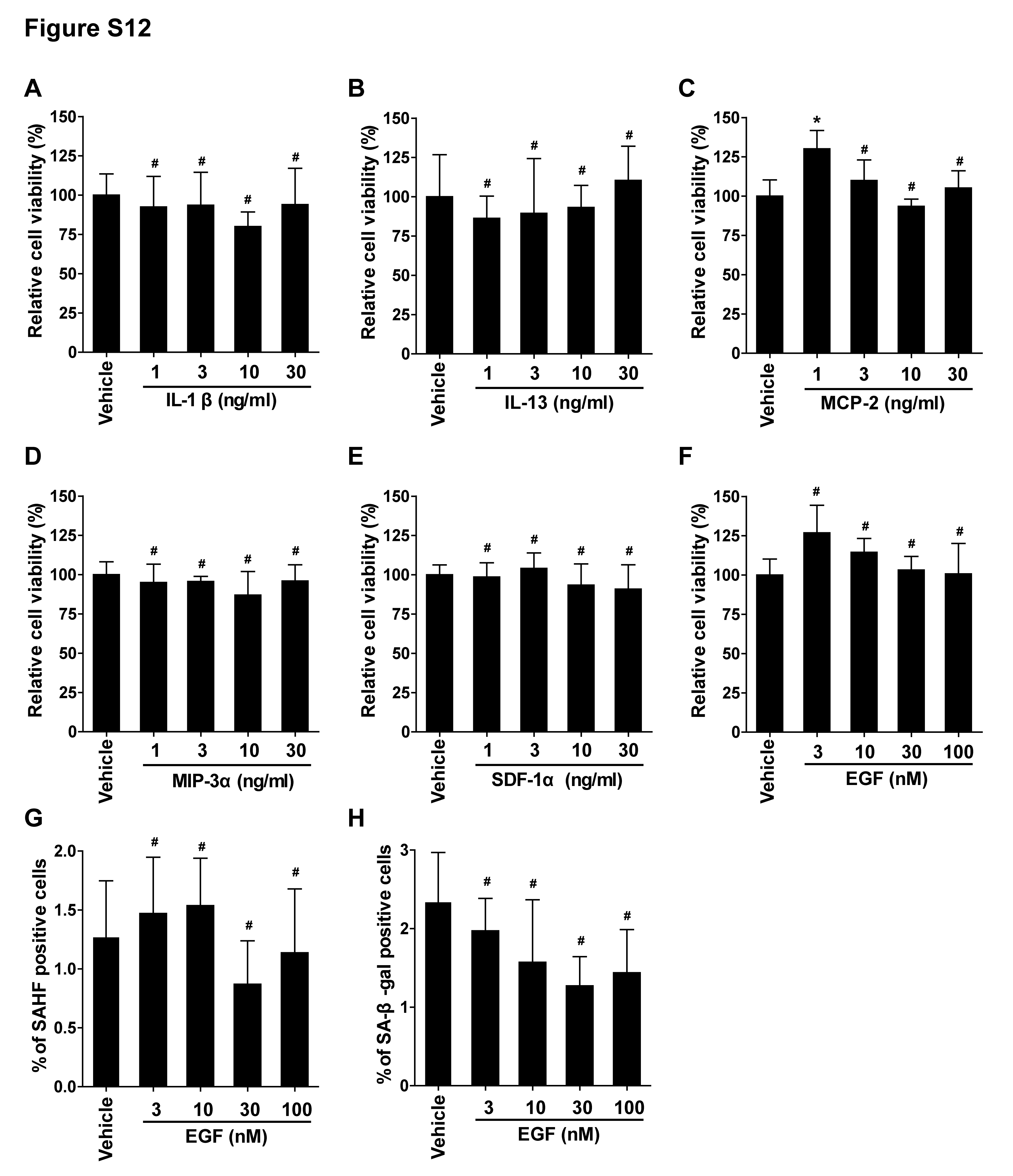

Supplement: Supplementary file 12 — Figure S12 [file ACEL-19-e13145-s012.tif]

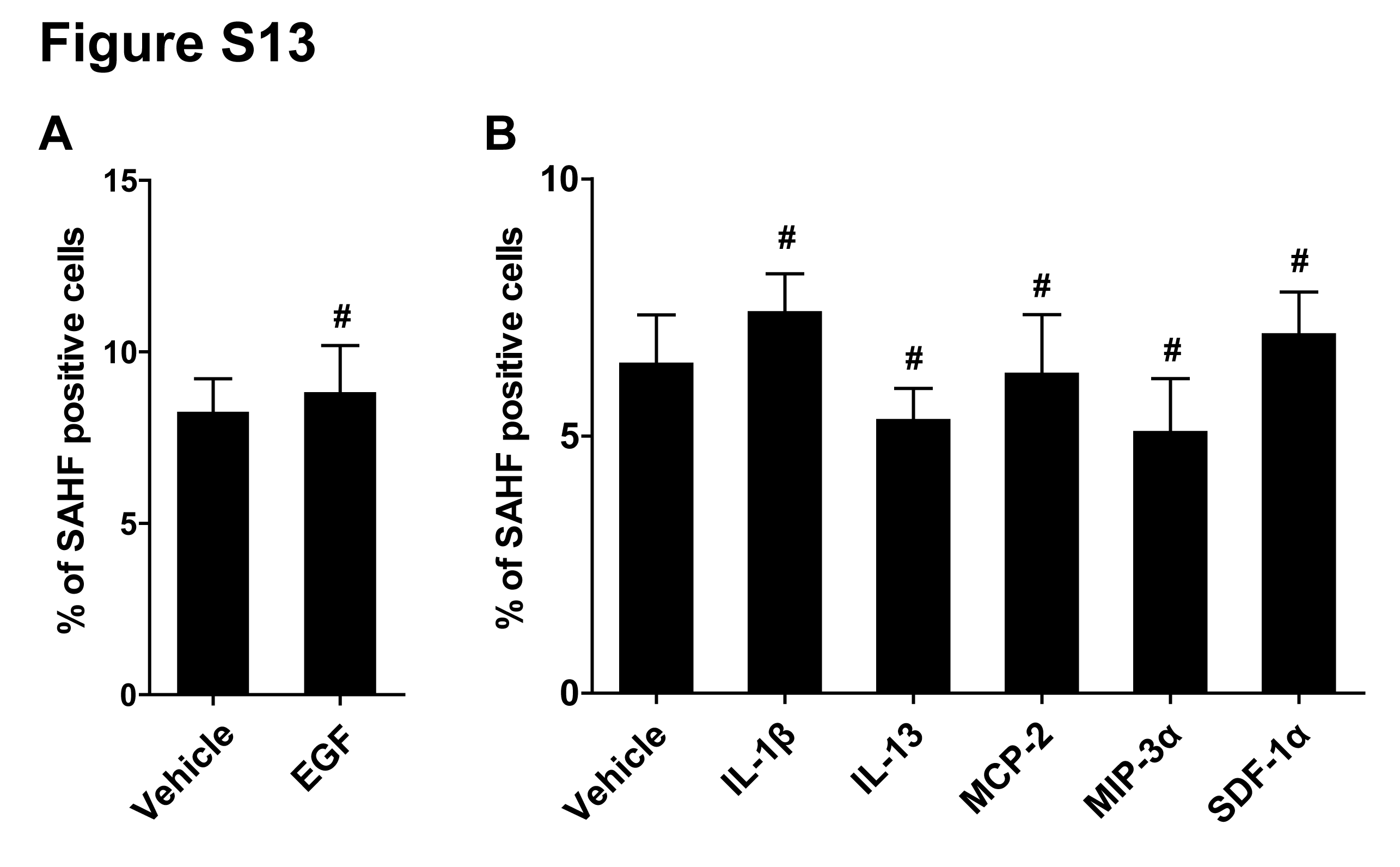

Supplement: Supplementary file 13 — Figure S13 [file ACEL-19-e13145-s013.tif]
