# Supplementary material for: Activation of epidermal growth factor receptor signaling mediates cellular senescence induced by certain pro‐inflammatory cytokines
Source: Aging Cell. 2020 Apr 22;19(5):e13145. doi: 10.1111/acel.13145 (PMC7253070; doi:10.1111/acel.13145)
Supplement: Supplementary file 14 — Table S1 [file ACEL-19-e13145-s014.doc]

**Supplementary Table 1. Antibodies used in this study.**

| **Targeted protein** | **Source** | **Company** | **Application** | **Dilution ratio** |
| --- | --- | --- | --- | --- |
| phospho-Histone H2A.X(Ser139) | rabbit | Cell Signaling Technology | Western blot | 1:1000 |
| 53BP1 | rabbit | BETHYL | Immunofluorescence & Western blot | 1:1000 |
| BrdU | mouse | BD | Immunofluorescence | 1:5 |
| p21 | rabbit | Santa Cruz | Western blot | 1:1000 |
| p16 | mouse | BD | Western blot | 1:1000 |
| p53 | mouse | Millipore | Western blot | 1:1000 |
| phospho-p53(Ser15) | rabbit | Cell Signaling Technology | Western blot | 1:1000 |
| phospho-EGFR（Tyr1068） | rabbit | Cell Signaling Technology | Western blot | 1:1000 |
| EGFR | rabbit | Ruiying Biological | Western blot | 1:1000 |
| ERK1/2 | rabbit | Ruiying Biological | Western blot | 1:1000 |
| phospho-ERK1/2(Thr202/Tyr204) | rabbit | Cell Signaling Technology | Western blot | 1:1000 |
| phospho-p38(Tyr182) | mouse | Santa Cruz | Western blot | 1:1000 |
| p38 | rabbit | Ruiying Biological | Western blot | 1:1000 |
| BRaf | mouse | Santa Cruz | Western blot | 1:1000 |
| β-actin | rabbit | Cell Signaling Technology | Western blot | 1:1000 |
| Goat anti-mouse Alexa-fluor 594 | goat | Life Technologies | Immunofluorescence | 1:1000 |
| Goat anti-rabbit Alexa-fluor 488 | goat | Life Technologies | Immunofluorescence | 1:1000 |
| Goat anti-Mouse IgG(H+L)HRP | goat | Jackson ImmunoResearch | Western blot | 1:5000 |
| Goat anti-Rabbit IgG(H+L)HRP | goat | Jackson ImmunoResearch | Western blot | 1:5000 |
