# Supplementary material for: Activation of epidermal growth factor receptor signaling mediates cellular senescence induced by certain pro‐inflammatory cytokines
Source: Aging Cell. 2020 Apr 22;19(5):e13145. doi: 10.1111/acel.13145 (PMC7253070; doi:10.1111/acel.13145)
Supplement: Supplementary file 15 — Table S2 [file ACEL-19-e13145-s015.doc]

**Supplementary Table 2. shRNAs sequences used in this study.**

| **Name** | **Sequences (5’to 3’)** |
| --- | --- |
| shEGFR-1 | CCGGCGCAAAGTGTGTAACGGAATACTCGAGTATTCCGTTACACACTTTGCGTTTTTTG |
| shEGFR-2 | CCGGCTGTGCAGAATCCTGTCTATCCTCGAGGATAGACAGGATTCTGCACAGTTTTTTG |
| shBRaf-1 | CCGGCTCAGTAAGGTACGGAGTAACCTCGAGGTTACTCCGTACCTTACTGAGTTTTTTG |
| shBRaf-2 | CCGGCCGCTGTCAAACATGTGGTTACTCGAGTAACCACATGTTTGACAGCGGTTTTT |
| shErk1/2-1 | CCGGCGTGCTCCACCGAGATCTAAACTCGAGTTTAGATCTCGGTGGAGCACGTTTTTG |
| shErk1/2-2 | CCGGGACAGACATCTCTGCACCCTGCTCGAGCAGGGTGCAGAGATGTCTGTCTTTTTTG |
