# Supplementary material for: Activation of epidermal growth factor receptor signaling mediates cellular senescence induced by certain pro‐inflammatory cytokines
Source: Aging Cell. 2020 Apr 22;19(5):e13145. doi: 10.1111/acel.13145 (PMC7253070; doi:10.1111/acel.13145)
Supplement: Supplementary file 16 — Table S3 [file ACEL-19-e13145-s016.doc]

**Supplementary Table 3. Primers for the qRT-PCR experiments.**

| **Targeted gene** | **Up-stream (5’-3’)** | **Down-stream (5’-3’)** |
| --- | --- | --- |
| GRO-α | gtccgtggccactgaact | ggggatgcaggattgaggc |
| IL-6 | agacagccactcacctcttc | tttcaccaggcaagtctcct |
| IL-8 | ttctgcagctctgtgtgaag | ccagttttccttggggtcca |
| MMP-3 | GTTCCGCCTGTCTCAAGATGA | GGGACAGGTTCCGTGGGTA |
| β-actin | AGAGCTACGAGCTGCCTGAC | AGCACTGTGTTGGCGTACAG |
