# Supplementary material for: Activation of epidermal growth factor receptor signaling mediates cellular senescence induced by certain pro‐inflammatory cytokines
Source: Aging Cell. 2020 Apr 22;19(5):e13145. doi: 10.1111/acel.13145 (PMC7253070; doi:10.1111/acel.13145)
Supplement: Supplementary file 17 — Table S4 [file ACEL-19-e13145-s017.docx]

**Supplementary Table 4. Pro-inflammatory cytokines used in screening.**

| **Cytokines** | **Chemokine** | **Others** |
| --- | --- | --- |
| IL-1β | Eotaxin-3 | bFGF |
| IL-6 | GRO-α | GM-CSF |
| IL-7 | IL-8 | IGF-BP7 |
| IL-13 | MCP-2 | KGF |
| IL-15 | MCP-3 | MMP-3 |
| TGF-β1 | MIP-1α | OPG |
|  | MIP-3α | VEGF |
|  | SDF-1α |  |
