# Supplementary material for: Activation of epidermal growth factor receptor signaling mediates cellular senescence induced by certain pro‐inflammatory cytokines
Source: Aging Cell. 2020 Apr 22;19(5):e13145. doi: 10.1111/acel.13145 (PMC7253070; doi:10.1111/acel.13145)
Supplement: Supplementary file 18 — Table S5 [file ACEL-19-e13145-s018.docx]

**Supplementary Table 5. Screening of SASP factors on IMR90 cells.**

| **Factors** | **SAHF positive** | | | | **β-gal positive** | | | | **If double positive** |
| --- | --- | --- | --- | --- | --- | --- | --- | --- | --- |
|  | **Control (%)**  **(a)** | **Highest (%)**  **(b)** | **Highest relative level**  **(b/a)** | **If>2.5** | **Control (%)**  **(a)** | **Highest (%)**  **(b)** | **Highest relative level**  **(b/a)** | **If>2.5** |  |
| **bFGF** | 7.7 | 9.7 | 1.3 | N | 4.4 | 6.7 | 1.5 | N | N |
| **Eotaxin-3** | 6.2 | 13.7 | 2.2 | N | 5.5 | 7.6 | 1.4 | N | N |
| **GM-CSF** | 8.3 | 11.4 | 1.4 | N | 4.5 | 6.4 | 1.4 | N | N |
| **IGF-BP7** | 6.3 | 8.8 | 1.4 | N | 4.3 | 9.7 | 2.2 | N | N |
| **IL-7** | 4.7 | 6.0 | 1.3 | N | 4.8 | 7.8 | 1.6 | N | N |
| **IL-15** | 5.3 | 7.7 | 1.4 | N | 4.3 | 10.3 | 2.4 | N | N |
| **MIP-1α** | 6.1 | 14.1 | 2.3 | N | 5.5 | 13.2 | 2.4 | N | N |
| **MMP-3** | 3.7 | 13.7 | 3.7 | Y | 6.6 | 13.0 | 2.0 | N | N |
| **OPG** | 8.7 | 12.0 | 1.4 | N | 4.6 | 5.5 | 1.2 | N | N |
| **VEGF** | 5.1 | 6.2 | 1.2 | N | 3.9 | 5.5 | 1.4 | N | N |
| **GRO-α** | 4.3 | 20.8 | 4.9 | Y | 4.3 | 17.3 | 4.0 | Y | Y |
| **IL-1β** | 7.8 | 20.9 | 2.7 | Y | 4.4 | 14.7 | 3.3 | Y | Y |
| **IL-6** | 5.5 | 18.0 | 3.2 | Y | 4.3 | 16.4 | 3.8 | Y | Y |
| **IL-8** | 5.6 | 20.3 | 3.6 | Y | 4.3 | 15.3 | 3.5 | Y | Y |
| **IL-13** | 5.2 | 17.0 | 3.3 | Y | 4.4 | 14.9 | 3.4 | Y | Y |
| **KGF** | 6.4 | 23.2 | 3.6 | Y | 4.5 | 16.6 | 3.7 | Y | Y |
| **MCP-2** | 4.6 | 20.1 | 4.4 | Y | 4.6 | 16.2 | 3.5 | Y | Y |
| **MCP-3** | 4.1 | 15.5 | 3.7 | Y | 4.4 | 13.8 | 3.1 | Y | Y |
| **MIP-3α** | 5.1 | 23.5 | 4.6 | Y | 3.2 | 15.2 | 4.7 | Y | Y |
| **SDF-1α** | 4.5 | 21.7 | 4.9 | Y | 4.2 | 15.8 | 3.7 | Y | Y |
| **TGF-β1** | 9.2 | 25.4 | 2.7 | Y | 4.5 | 14.3 | 3.2 | Y | Y |

**Data were extracted from Figure S1 and S2. ‘a’ means the positive ratio of the control group. ‘b’ means the highest positive ratio reached in a cytokine-treated group. A cytokine is marked in blue if single positive and red if double positive.**
