# Supplementary material for: Activation of epidermal growth factor receptor signaling mediates cellular senescence induced by certain pro‐inflammatory cytokines
Source: Aging Cell. 2020 Apr 22;19(5):e13145. doi: 10.1111/acel.13145 (PMC7253070; doi:10.1111/acel.13145)
Supplement: Supplementary file 19 — Table S6 [file ACEL-19-e13145-s019.doc]

| **Factors** | **SAHF positive** | | | | **β-gal positive** | | | | **If double positive** |
| --- | --- | --- | --- | --- | --- | --- | --- | --- | --- |
|  | **Control (%)**  **(a)** | **Highest (%)**  **(b)** | **Highest relative level**  **(b/a)** | **If>2.5** | **Control (%)**  **(a)** | **Highest (%)**  **(b)** | **Highest relative level**  **(b/a)** | **If>2.5** |  |
| **bFGF** | 5.6 | 6.0 | 1.1 | N | 3.5 | 4.3 | 1.2 | N | N |
| **Eotaxin-3** | 7.2 | 7.2 | 1.0 | N | 5.6 | 6.1 | 1.1 | N | N |
| **GM-CSF** | 6.3 | 6.7 | 1.1 | N | 5.4 | 6.8 | 1.3 | N | N |
| **IGF-BP7** | 4.5 | 4.1 | 0.9 | N | 5.5 | 5.0 | 0.9 | N | N |
| **IL-7** | 7.3 | 9.5 | 1.3 | N | 6.5 | 12.9 | 2.0 | N | N |
| **IL-15** | 4.7 | 13.3 | 2.8 | Y | 4.3 | 13.2 | 3.1 | Y | Y |
| **MIP-1α** | 5.2 | 8.0 | 1.5 | N | 5.3 | 8.1 | 1.5 | N | N |
| **MMP-3** | 5.4 | 9.6 | 1.8 | N | 4.0 | 6.5 | 1.6 | N | N |
| **OPG** | 3.8 | 6.8 | 1.8 | N | 6.5 | 5.7 | 0.9 | N | N |
| **VEGF** | 4.7 | 6.1 | 1.3 | N | 4.5 | 4.1 | 0.9 | N | N |
| **GRO-α** | 4.6 | 5.9 | 1.3 | N | 7.5 | 10.7 | 1.4 | N | N |
| **IL-1β** | 5.7 | 19.7 | 3.5 | Y | 4.9 | 15.1 | 3.1 | Y | Y |
| **IL-6** | 5.3 | 16.3 | 3.1 | Y | 3.6 | 12.2 | 3.4 | Y | Y |
| **IL-8** | 6.4 | 16.7 | 2.6 | Y | 4.2 | 5.9 | 1.4 | N | N |
| **IL-13** | 5.4 | 15.4 | 2.9 | Y | 4.4 | 17.6 | 4.0 | Y | Y |
| **KGF** | 5.3 | 11.1 | 2.1 | N | 4.3 | 10.1 | 2.3 | N | N |
| **MCP-2** | 5.1 | 15.5 | 3.0 | Y | 3.4 | 15.9 | 4.7 | Y | Y |
| **MCP-3** | 6.7 | 25.6 | 3.8 | Y | 4.0 | 15.1 | 3.8 | Y | Y |
| **MIP-3α** | 7.7 | 20.2 | 2.6 | Y | 5.0 | 15.0 | 3.0 | Y | Y |
| **SDF-1α** | 3.8 | 14.6 | 3.8 | Y | 4.9 | 13.2 | 2.7 | Y | Y |
| **TGF-β1** | 5.9 | 18.7 | 3.2 | Y | 5.5 | 6.7 | 1.2 | N | N |

**Supplementary Table 6. Screening of SASP factors on HUVECs (Donor 1).**

**Note: ‘a’ means the positive ratio of the control group. ‘b’ means the highest positive ratio reached in a cytokine-treated group. A cytokine is marked in blue if single positive and red if double positive.**
