# Supplementary material for: Activation of epidermal growth factor receptor signaling mediates cellular senescence induced by certain pro‐inflammatory cytokines
Source: Aging Cell. 2020 Apr 22;19(5):e13145. doi: 10.1111/acel.13145 (PMC7253070; doi:10.1111/acel.13145)
Supplement: Supplementary file 22 — Table S9 [file ACEL-19-e13145-s022.doc]

**Supplementary Table 9. The effect of Cetuximab on senescence-inducing pro-inflammatory cytokines.**

| **Factors** | **SAHF positive** | | | | | **β-gal positive** | | | | |
| --- | --- | --- | --- | --- | --- | --- | --- | --- | --- | --- |
|  | **Vehicle-**  **Vehicle** **(%)**  **(a)** | **Factor-**  **Vehicle (%)**  **(b)** | **Factor-**  **Cetuximab (%)**  **(c)** | **Relative level** | | **Vehicle-**  **Vehicle (%)**  **(a)** | **Factor-**  **Vehicle (%)**  **(b)** | **Factor-**  **Cetuximab (%)**  **(c)** | **Relative level** | |
|  |  |  |  | **b/a** | **c/a** |  |  |  | **b/a** | **c/a** |
| **IL-1β** | 5.5 | 19.5 | 8.1 | 3.5 | 1.5 | 4.8 | 14.2 | 7.0 | 3.0 | 1.5 |
| **IL-13** | 5.0 | 15.6 | 5.3 | 3.1 | 1.1 | 4.8 | 14.1 | 8.2 | 2.9 | 1.7 |
| **MCP-2** | 5.6 | 23.2 | 6.1 | 4.1 | 1.1 | 6.2 | 17.6 | 6.7 | 2.8 | 1.1 |
| **MCP-3** | 4.5 | 12.9 | 11.9 | 2.9 | 2.6 | 5.5 | 16.2 | 14.8 | 3.0 | 2.7 |
| **MIP-3α** | 5.9 | 21.7 | 7.5 | 3.7 | 1.3 | 5.7 | 16.3 | 4.3 | 2.9 | 0.8 |
| **SDF-1α** | 5.2 | 18.9 | 6.2 | 3.6 | 1.2 | 5.4 | 15.9 | 7.1 | 2.9 | 1.3 |

**Data were extracted from Figure S3. Note: a, b, and c represents the positive ratios in each group with the indicated treatments, respectively.**

**The relative level was calculated using the formula in the table.**
