# Supplementary material for: Activation of epidermal growth factor receptor signaling mediates cellular senescence induced by certain pro‐inflammatory cytokines
Source: Aging Cell. 2020 Apr 22;19(5):e13145. doi: 10.1111/acel.13145 (PMC7253070; doi:10.1111/acel.13145)
Supplement: Supplementary file 23 — Table S10 [file ACEL-19-e13145-s023.doc]

**Supplementary Table 10. The effect of gefitinib on senescence-inducing pro-inflammatory cytokines.**

| **Factors** | **SAHF positive** | | | | | **β-gal positive** | | | | |
| --- | --- | --- | --- | --- | --- | --- | --- | --- | --- | --- |
|  | **Vehicle-**  **Vehicle** **(%)**  **(a)** | **Factor-**  **Vehicle (%)**  **(b)** | **Factor-**  **Gefitinib (%)**  **(c)** | **Relative level** | | **Vehicle-**  **Vehicle (%)**  **(a)** | **Factor-**  **Vehicle (%)**  **(b)** | **Factor-**  **Gefitinib (%)**  **(c)** | **Relative level** | |
|  |  |  |  | **b/a** | **c/a** |  |  |  | **b/a** | **c/a** |
| **IL-1β** | 4.1 | 16.2 | 6.6 | 4.0 | 1.6 | 3.0 | 10.9 | 6.5 | 3.6 | 2.2 |
| **IL-13** | 4.3 | 16.6 | 10.9 | 3.9 | 2.5 | 3.3 | 10.0 | 6.3 | 3.0 | 1.9 |
| **MCP-2** | 3.9 | 16.9 | 7.5 | 4.3 | 1.9 | 3.2 | 13.1 | 5.7 | 4.1 | 1.8 |
| **MCP-3** | 5.6 | 15.3 | 16.2 | 2.7 | 2.9 | 3.8 | 13.2 | 12.5 | 3.5 | 3.3 |
| **MIP-3α** | 5.4 | 14.7 | 8.5 | 2.7 | 1.6 | 3.9 | 12.1 | 7.1 | 3.1 | 1.8 |
| **SDF-1α** | 3.9 | 15.9 | 4.0 | 4.1 | 1.0 | 3.6 | 13.8 | 5.7 | 3.8 | 1.6 |

**Data were extracted from Figure S4. Note: a, b, and c represents the positive ratios in each group with the indicated treatments, respectively.**

**The relative level was calculated using the formula in the table.**
