# Supplementary material for: Activation of epidermal growth factor receptor signaling mediates cellular senescence induced by certain pro‐inflammatory cytokines
Source: Aging Cell. 2020 Apr 22;19(5):e13145. doi: 10.1111/acel.13145 (PMC7253070; doi:10.1111/acel.13145)
Supplement: Supplementary file 24 — Supplementary Material [file ACEL-19-e13145-s024.doc]

**Supplementary Figures**

**Figure S1. Screening of pro-inflammatory cytokines on IMR90 cells by SAHF assay.** A, percentages of SAHF-positive cells induced by ‘negative’ cytokines. B, percentages of SAHF-positive cells induced by ‘positive’ cytokines. Data indicate the mean values calculated from three independent experiments (±SD). A factor was defined as ‘positive’ if it increased the percentages of positive cells more than 2.5-fold compared to the vehicle group in a dose-dependent manner.

**Figure S2. Screening of pro-inflammatory cytokines on IMR90 cells by SA-β-gal assay.** A, percentages of SA-β-gal-positive cells induced by ‘negative’ cytokines. B, percentages of SA-β-gal-positive cells induced by ‘positive’ cytokines. Data indicate the mean values calculated from three independent experiments (±SD). A factor was defined as ‘positive’ if it increased the percentages of positive cells more than 2.5-fold compared to the vehicle group in a dose-dependent manner.

**Figure S3. The effect of cetuximab treatment on senescence-inducing cytokines.** A, percentages of SAHF-positive cells with different treatment as indicated. B, percentages of SA-β-gal-positive cells with different treatments as indicated. Data indicate the mean values calculated from three independent experiments (±SD). The cytokines were applied at their optimal concentrations individually. Cetuximab was added at 10 μg/ml to the medium and applied together with the cytokines.

**Figure S4. The effect of gefitinib treatment on senescence-inducing cytokines.** A, percentages of SAHF-positive cells with different treatment as indicated. B, percentages of SA-β-gal-positive cells with different treatments as indicated. Data indicate the mean values calculated from three independent experiments (±SD). The cytokines were applied at their optimal concentrations individually. Gefitinib was applied at 100 ng/ml in medium together with the cytokines.

**Figure S5. The effect of EGFR knockdown on senescence-inducing cytokines.** A, percentages of SAHF-positive cells with different treatment as indicated. B, percentages of SA-β-gal-positive cells with different treatments as indicated. Data indicate the mean values calculated from three independent experiments (±SD). The cytokines were applied at their optimal concentrations individually. IMR90 cells were infected with vector or shRNA-expressing virus and selected for 2 days before being treated with cytokines.

**Figure S6. IL-1β or SDF-1α treatment did not increase EGF secretion from IMR90 cells.** Cells were treated with vehicle, IL-1β, or SDF-1α at their optimal concentrations for 2 days. The conditioned media were then collected and tested using an ELISA kit for EGF detection. The results were normalized against the average of the vehicle group. Data indicate the mean values calculated from three independent experiments (±SD).

**Figure S7. FACS analysis of apoptotic cells with different treatments.** Representative flow cytometry plots of IMR90 cells treated with vehicle (A), EGF (B, C) at the indicated concentration, 10 μM of cisplatin (D) for 3 days, or heating for 5 minutes at 50℃ (E), before staining with annexin V-FITC/propidium iodide and analysis for cell apoptosis distribution. F, the above experimental results were analyzed for the presence of annexin V (+)/PI (-) (early apoptosis) and annexin V (+)/PI (+) (late apoptosis) and the sum of early and late apoptosis was calculated. Data shown are the mean values (±SD) from three independent experiments.

**Figure S8. EGF treatment increased active Ras in HUVECs.** A and B, HUVECs were treated with EGF at the indicated concentrations for 10 minutes before GST-Raf-RBD pull-down assays. Levels of GTP-bound Ras were determined. The experiments were repeated twice.

**Figure S9. The combinational treatment using EGF and cytokines.** A, percentages of SAHF-positive cells in the groups with different treatments as indicated. B, percentages of SA-β-gal-positive cells. Cytokines and EGF were applied together at the beginning of the experiments and EGF was replenished 24 hours later. After another 24 hours, the cells were applied to SAHF- and SA-β-gal staining. Data shown are the mean values (±SD) from three independent experiments. ***, *P* < 0.001, # > 0.05, compared to EGF-vehicle group.

**Figure S10. The activation of EGFR induces the secretion of IL-8 and MMP-3 in IMR90 cells.** A, the mRNA levels of GRO-α, IL-6, IL-8, and MMP-3 in IMR90 cells with or without EGF treatment (100 nM). B, the relative secretory levels of IL-8 and MMP-3 in the conditioned media of IMR90 cells with or without EGF treatment (100 nM). C and D, the relative secretory levels of IL-8 and MMP-3 in the conditioned media of IMR90 cells with or without cytokine treatment at their optimal concentrations. Data indicate the mean values calculated from three independent experiments (±SD). *, *P* < 0.05; **, *P* < 0.01; ***, *P* < 0.001; Compared to the vehicle group. §§, *P* < 0.01; §§§, *P* < 0.001; Compared to the IL-1β group.

**Figure S11. The effects of cytokines and EGF on MGC-803 cells.** A-E, the viabilities of cells treated with or without cytokines at different concentrations for 2 days. F, the viabilities of the cells treated with or without EGF at different concentrations for 3 days. G and H, percentages of SAHF and SA-β-gal-positive cells in F. Data indicate the mean values calculated from three independent experiments (±SD). #, *P* ≥ 0.05; *, *P* < 0.05; **, *P* < 0.01; ***, *P* < 0.001; compared to the vehicle group.

**Figure S12. The effects of cytokines and EGF on PC-9 cells.** A-E, the viabilities of cells treated with or without cytokines at different concentrations for 2 days. F, the viabilities of the cells treated with or without EGF at different concentrations for 3 days. G and H, percentages of SAHF- and SA-β-gal-positive cells in F. Data indicate the mean values calculated from three independent experiments (±SD). #, *P* ≥ 0.05; *, *P* < 0.05; **, *P* < 0.01; ***, *P* < 0.001; compared to the vehicle group.

**Figure S13. The effects of cytokines and EGF on the quiescent cells.** A, the percentages of SAHF-positive cells in IMR90 cells with or without EGF treatment (100 nM). Quiescent IMR90 cells were established by contact inhibition and low FBS (0.5%). B, the percentages of SAHF-positive cells in IMR90 cells with or without cytokines treatment at their optimal concentrations. Data indicate the mean values calculated from three independent experiments (±SD). #, *P* ≥ 0.05; compared to the vehicle group.
